# Protocol for an international multicenter, prospective, observational, non-competitive, study to validate and optimise prediction models of 90-day and 1-year allograft failure after liver transplantation: The global IMPROVEMENT Study

**DOI:** 10.1007/s13304-025-02078-4

**Published:** 2025-03-27

**Authors:** Alfonso W. Avolio, Gabriele Spoletini, Umberto Cillo, Kristopher Croome, Gabriel Oniscu, Patrizia Burra, Martin De Santibanes, Hiroto Egawa, Mikel Gastaca, Zhiyong Guo, Quirino Lai, Paulo N. Martins, Wojciech G. Polak, Cristiano Quintini, Mohamed Rela, Gonzalo Sapisochin, Julio Wiederkehr, Riccardo Pravisani, Deniz Balci, Ian Leipnitz, Ilka Boin, Felix Braun, Lucio Caccamo, Stefania Camagni, Amedeo Carraro, Matteo Cescon, Zhishui Chen, Olga Ciccarelli, Luciano De Carlis, Deng Feiwen, Fabrizio Di Benedetto, Burcin Ekser, Giuseppe Maria Ettorre, Marta Garcia-Guix, Davide Ghinolfi, Michal Grat, Salvatore Gruttadauria, John Hammond, Zemin Hu, Sunhawit Junrungsee, Michael Lesurtel, Jean Yves Mabrut, Daniel Maluf, Vincenzo Mazzaferro, Gilberto Mejia, Artem Monakhov, Bunthoon Noonthasoot, Silvio Nadalin, Brian M. Nguyen, Nguyen Quang Nghia, Madhukar Patel, Thamara Perera, Marcos Vinicius Perini, Carlo Pulitano, Renato Romagnoli, Ephrem Salame, Gupta Subhash, Surendran Sudhindran, Takashi Ito, Francesco Tandoi, Giuliano Testa, Timucin Taner, Giuseppe Tisone, Giovanni Vennarecci, Marco Vivarelli, Diana Giannarelli, Tina Pasciuto, Marco Maria Pascale, Vatche Agopian, and the global IMPROVEMENT study group

**Affiliations:** 1 General Surgery and Transplantation Unit, Fondazione Policlinico Universitario Agostino Gemelli IRCCS, Rome, Italy; 2 General Surgey 2 Hepatobiliopancreatic Surgery and Liver Transplan Unit, Azienda Ospedaliera Universitaria, Padua, Italy; 3 Division of Transplant Surgery, Department of Transplant, Mayo Clinic, Jacksonville, FL USA; 4 Division of Transplantation, Clintec Karolinska University Hospital, Stockholm, Sweden; 5 Multivisceral Transplant Unit, Azienda Ospedaliera Universitaria, Padua, Italy; 6 Department of Hepato-Biliary, Pancreatic Surgery & Liver Transplant Unit, Hospital Italiano de Buenos Aires, Buenos Aires, Argentina; 7 Department of Surgery, Institute of Gastroenterology, Tokyo Women’s Medical University, Shizuoka, Japan; 8 Unidad de Cirugía Hepatobiliar y Trasplante Hepático, Hospital Universitario Cruces-Bilbao, Bilbao, Spain; 9 Organ Transplant Center, The First Affiliated Hospital of Sun Yat-Sen University, Guangzhou, China; 10 Hepato-Bilio-Pancreatic and Liver Transplant Unit, Department of Surgery, Sapienza University, Rome, Italy; 11 Transplant Division, Dept of Surgery, University of Massachusetts, Worcester, MA USA; 12 Department of Surgery, Division of HPB and Transplant Surgery, Erasmus MC Transplant Institute, University MC Rotterdam, Rotterdam, the Netherlands; 13 Department of Liver Transplantation, Cleveland Clinic Abu Dhabi, Abu Dhabi, United Arab Emirates; 14 Institute of Liver Disease and Transplantation, Dr Rela Institute and Medical center, Bharath Institute of Higher Education and Research, Chennai, India; 15 Multiorgan Transplantation, Toronto General Hospital, Toronto, Canada; 16 Liver Transplant Division, Hospital Santa Isabel, Blumenau, Brazil; 17 Liver-Kidney Transplant Unit, Università di Udine – ASUFC, Udine, Italy; 18 Liver Transplantation Unit, Department of General Surgery, Faculty of Medicine, Ankara University, Ankara, Turkey; 19 Liver Transplant Unit, University of Auckland, Auckland, New Zealand; 20 Liver Transplantation Unit, University of Campinas-UNICAMP, S. Paolo, Brazil; 21 Department of General, Visceral-, Thoracic-, Transplant- and Pediatric-Surgery, University Hospital Schleswig-Holstein, Campus Kiel, Kiel, Germany; 22 General and Liver Transplant Surgery, Fondazione IRCCS Cà Grande Ospedale Maggiore Policlinico Milano, Milan, Italy; 23 Department of Organ Failure and Transplantation, ASST Papa Giovanni XXIII, Bergamo, Italy; 24 Liver Transplant Unit, University Hospital Trust of Verona, Verona, Italy; 25 General Surgery and Transplant Unit, Azienda Ospedaliero-Universitaria di Bologna, Policlinico di Sant’Orsola, Bologna, Italy; 26 Laboratory of Organ Transplantation, Institute of Organ Transplantation, Tongji Hospital, Wuhan, China; 27 Service de Chirurgie et Transplantation Abdominal, Cliniques Universtaires Saint-Luc, Louvein, Belgium; 28 General Surgery and Abdominal Transplantation Unit, University of Milano-Bicocca and Niguarda-CàGranda Hospital, Milan, Italy; 29 Department of Hepatopancreas Surgery, Foshan First People’s Hospital, Foshan, China; 30 Hepato-Pancreato-Biliary Surgery and Liver Transplantation Unit, University of Modena and Reggio Emilia, Modena, Italy; 31 Division of Transplant Surgery, Department of Surgery, Indiana University School of Medicine, Indianapolis, IN USA; 32 Department of General Surgery and Transplantation Unit, A.O. San Camillo-Forlanini, Rome, Italy; 33 Division of Hepatobiliary and Liver Transplantation, Department of Surgery, University of Barcelona, Barcelona, Spain; 34 Division of Hepatic Surgery and Liver Transplantation, University of Pisa Hospital, Pisa, Italy; 35 Transplant and Liver Surgery, Public Central Teaching Hospital, Medical University of Warsaw, Warsaw, Poland; 36 Abdominal Transplantation, IRCCS ISMETT – UPMC, Palermo, Italy; 37 HPB and Transplant Surgery, Newcastle Hospital NHS Foundation Trust, Newcastle, UK; 38 General Surgery 1, Zhongshan People’s Hospital, Zhongshan, China; 39 Division of Hepato-Biliary-Pancreas Surgery, Chiang Mai University, Chiang Mai, Thailand; 40 Department of HPB Surgery & Liver Transplantation, Beaujon Hospital, Université Paris Cité, Paris, France; 41 Department of General Surgery and Liver Transplantation, Croix-Rousse University Hospital, Hospices Civils de Lyon, Lyon, France; 42 Department of Surgery, University of Maryland, Baltimore, MD USA; 43 General Surgery and Liver Transplantation Unit, University of Milan and National Cancer Institute, IRCCS, Milan, Italy; 44 Transplant Surgery, Fundacion CardioInfantil, Bogotà, Colombia; 45 Surgical Department #2 (Liver Transplantation), National Medical Research Center of Transplantation and Artificial Organs named after V.I. Shumakov, Moscow, Russia; 46 Department of Surgery, Faculty of Medicine, Chulalongkorn University, Bangkok, Thailand; 47 Department of General, Visceral and Transplant Surgery, Universitätsklinik Tübingen, Tubingen, Germany; 48 MedStar Georgetown Transplant Institute, MedStar Georgetown University Hospital, Georgetown, Washington, DC USA; 49 Center of Organ Transplantation, Viet Duc University Hospital, Hanoi, Vietnam; 50 Liver Transplantation Unit, University of Texas Southwestern Medical Center, Dallas, TX USA; 51 Transplant Surgery, Queen Elizabeth Hospital, Birmingham, UK; 52 Liver Transplantation Unit, Austin Hospital, Melbourne, VIC Australia; 53 Australian National Liver Transplantation Unit, Royal Prince Alfred Hospital, Faculty of Medicine and Health, University of Sydney, Sydney, NSW Australia; 54 General Surgery 2U, Liver Transplantation Center, Azienda Ospedaliero-Universitaria Città della Salute e della Scienza di Torino, Turin, Italy; 55 Department of Digestive, Hepatobiliary and Pancreatic Surgery, Regional University Hospital, Tours, France; 56 center for Liver and Biliary Science, Max Super Speciality Hospital Saket, New Delhi, India; 57 Dept of GI Surgery, Amrita Institute of Medical Sciences (Amrita Hospital), Kochi, India; 58 Dept of Surgery, Graduate School of Medicine, Kyoto University, Kyoto, Japan; 59 Hepatobiliary Surgery and Liver Transplantation, AOU Policlinico Consorziale di Bari, Bari, Italy; 60 Baylor Scott & White, All Saints Medical Center & Baylor University Medical Center, Ft. Worth & Dallas, TX USA; 61 Center for Transplantation and Clinical Regeneration, Mayo Clinic, Rochester, MN USA; 62 HPB and Transplant Unit, Department of Surgical Sciences, University of Rome Tor Vergata, Rome, Italy; 63 UOC Hepato-Biliary Surgery and Liver Transplant center, A.O.R.N.A. CARDARELLI, Naples, Italy; 64 Hepatobiliary and Abdominal Transplantation Surgery, Ancona Hospital, Ancona, Italy; 65 Dept Epidemiology and Biostatistics, Fondazione Policlinico Universitario Agostino Gemelli, IRCCS, Rome, Italy; 66 Hygiene Unit, University Department of Life Sciences and Public Health, Università Cattolica Del Sacro Cuore, Rome, Italy; 67 Research Core Facility Data Collection G-STeP, Fondazione Policlinico Universitario Agostino Gemelli IRCCS, Rome, Italy; 68 Division of Liver and Pancreas Transplantation, Department of Surgery, David Geffen School of Medicine at UCLA, Los Angeles, CA USA

**Keywords:** Liver transplant, Allograft failure, Retransplant, Outcome, Ischemic cholangiopathy, Primary dysfunction

## Abstract

More liver transplants (LT) are performed worldwide thanks to extended criteria donors (ECD). This is paralleled by a supposed increased risk of allograft failure (AF) at 90 and 365 days. This study has been designed to portray the LT practice worldwide and investigate models of AF prediction and the impact of risk mitigation strategies for further improving graft and patient outcomes. This is a multicenter, international, non-competitive, observational two segment study on consecutive LTs over two periods (2017–2019 and 2022–2024). A steering committee of LT experts defined the study protocol. The prospective segment will enroll 750 patients from 15 high-volume LT centers (50 per center), and the retrospective segment will enrol 4200 patients from 56 LT centers (75 per center). To provide a snapshot of the LT activity globally and to develop new algorithms for the timely prediction of AF at 90 and 365 days post-LT. The study also aims (1) to validate the existing predictive models and (2) to investigate the best time for re-transplantation, paying attention to the differences in AF and Ischemic cholangiopathy according to the donor types and mitigation strategies implemented in the various settings. Since the adoption of machine perfusion has increased in different proportions worldwide, models will be adjusted according to this parameter. Finally, retrospective and prospective data will be available for further stratifications and modelling according to the degree of decompensation at transplant, gender match, postoperative complications and their management. This protocol was approved by Fondazione Policlinico Universitario Agostino Gemelli IRCCS Ethics Committee (study ID: 4571) and the Institutional Review Board of the University of California, Los Angeles. The provisional study protocol was submitted to the main scientific international societies in the transplant field. Results will be published in international peer-reviewed journals and presented at congresses.

**Graphical Abstract:**

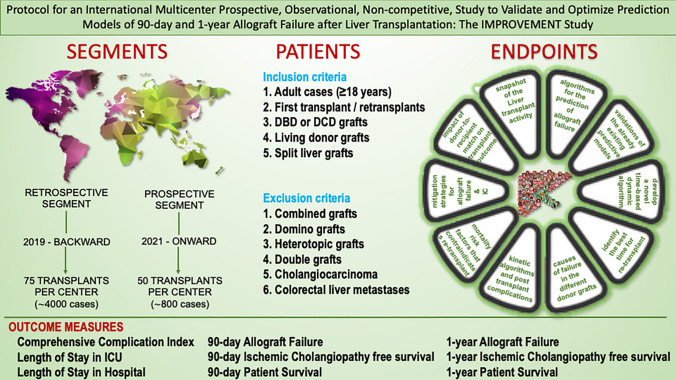

**Supplementary Information:**

The online version contains supplementary material available at 10.1007/s13304-025-02078-4.

## Introduction

During the last decade, the expansion of the donor pool using extended criteria (ECD) and donation after cardiocirculatory death donors (DCD) has resulted in heightened attention to allograft failure (AF) due to ischemia–reperfusion injury after liver transplantation (LT). The prompt diagnosis of AF is paramount to defining the indication for early retransplantation. Moreover, the grafts from ECD and DCD may develop early AF during the initial 90 days [1, 2] and delayed AF, primarily due to ischemic cholangiopathy [3]. LT from living donors (LDLT) is a growing procedure worldwide. However, the prevalence of steatosis in the general population is growing, too. A safety threshold for graft steatosis has yet to be entirely investigated, and the possible role of mitigation strategies to reduce the risk of AF in LDLT is a critical unmet need.

The evaluation times and the modalities to promptly identify AF are still the object of research. No consensus exists on the most accurate predictors and indicators of AF, neither on potential mitigation strategies [4–11]. In addition, ECD and DCD grafts are burdened by a higher incidence of ischemic cholangiopathy [12], which typically develops after 6–12 months, which is also a cause of AF and need for retransplantation. With the intent to further expand and optimize the donor pool, machine perfusion (MP) technologies are increasingly employed, allowing utilization of organs that have traditionally been discarded. Moreover, MP allows extended preservation times and assessment of graft viability, especially when considering organs with multiple risk factors (e.g., elderly donors, steatotic grafts, post-anoxic grafts, etc.). [13]

Multiple attempts have been made at evaluating the degree of organ damage and capability to recover function after LT. From the original definition of early allograft dysfunction (EAD) by Olthoff et al. [14, 15], through the model for early allograft function (MEAF) score by Pareja et al. [6] a new category of scores based on the kinetics of multiple parameters have recently emerged. Two scores, one developed in the United States at UCLA (L-GrAFT) [1] and one in Italy from a multicenter cohort (EASE) [2], allow the prediction of early AF with excellent C-statistics. Both were validated on multicentric external populations [16, 17]. L-GrAFT and EASE scores are calculated at day 10 after LT; furthermore, a modification of the L-GrAFT score, calculated at day 7, was subsequently developed and validated [17]. Both scores are also proposed for quantifying the degree of graft recovery in translational studies [18–20]. A synopsis of the characteristics of the different scores is displayed in Table 1. However, the prognostic role of pretransplant graft macrosteatosis, the differences among phenotypic patterns of various transplant types (DBD, DCD, and living donor grafts), as well as the detailed characterization of the dynamics that make retransplantation sustainable or contraindicated have not been investigated [21, 22]. Prospective studies evaluating these endpoints and further mitigation strategies to reduce graft-related risks are still awaited.

**Table 1:** Previous scores (object, endpoint, cutoff, number of factors utilized, number of entries, accuracy, discrimination power, strength and limitations)

	DRI (2006) [9]	EAD (2010) [5]	D-MELD (2011) [18]	New ET-DRI (2012) [19]	SOFT (2008) [20]	MEAF (2015) [6]	L-GrAFT (2018) [1]	L-GrAFT (2021) [15]	EASE (2020) [2]
Object of score	Donor quality	Graft quality	Donor–recipient match	Donor quality	Donor–recipient match	Graft recovery	Graft recovery	Graft recovery	Graft recovery
Origin of data	UNOS registry data	3 centers	20 centers	EURO TRANSPLANT registry data	UNOS	Monocentric	1 Center	7 Centers	16 Centers
*N* of patients (developing set)	20,023 1998–2002	297 2004–2005	3281 2002–2009	5723 2003–2007	21,673 2002–2006	1026			
Endpoint	Graft failure	Graft dysfunction	Graft failure/patient death	Graft failure	Patient death	Graft failure	Graft failure	Graft failure	Graft failure
Cutoff and % of predicted at cases	≥ 2 80 % 90 dd	4 75% at 180 dd	Failure: > 1628; 84% at 90 dd Death: > 1628; 86% at 90 dd	> 2 79% at 90 dd	> 40 –	≥ 8 70% at 90 dd	> 1.3 16% at 90 dd	> 1.3 16% at 90 dd	> 0 28% at 90 dd
Evaluation time in relation to LT	Intraoperative (CIT)	7 days	Few hours before LT	intraoperative	Intraoperative CIT	3 dd	10 dd	7 dd	10 dd
Donor	Yes	–	Yes	Yes	Yes	–	–	–	–
Recipient preoperative data	–	–	Yes	–	Yes	–	–	–	Yes
Intraoperative data	–	–	–	–	–	–	–	–	Yes
Transplant logistics	CIT	–	–	Yes	CIT	–	–	–	–
Post-operative data	–	Yes	–	–	–	Yes	Yes	Yes	Yes
Center volume stratification	–	–	–	–	–	–	–	–	yes
Number of variables	8	3	2	8*	19	3	4	4	7*
Total # of determinations	8	3	2	8	19	9	40	27	17
Accuracy at 90 days (derivation set when available)	Not reported	0.72^§^	0.70 and 0.64	0.63	0.69	Not reported	0.85	0.80	0.87
Accuracy at 90 days (validation set or external data set^$^)	0.57^$^	0.63^§$^	0.72 and 0.64	0.58 ^$^	–	0.73 ^$^	0.74	0.74	0.78
Discrimination between highest and lowest risk class					57%	26%			
Strengths	Diffusion		Match oriented		High discrimination	1rst kinetic method	High accuracy high discrimination	High accuracy high discrimination high accuracy in the validation set	High accuracy high discrimination
Limitations	Old, low accuracy, low discrimination		Intermediate accuracy		Intermediate accurac y	Low discrimination	Complexity of calculation (computer)	Complexity of calculation (computer)	Complexity (smartphones/web application) inferior accuracy in the validation set

Abbreviations. $, external data set; §, at 180 days

 Timely prediction of AF is pivotal to identifying patients who may potentially benefit from a rescue retransplant before severe complications develop. When massive cytolysis and clear signs of liver failure occur within the first 2–4 days after LT, the indication for retransplantation is clear. Diversely, the decision to retransplant a patient with a milder degree of AF (also called dysfunction) is more challenging, especially after the first 5–10 postoperative days. AF results from a complex interplay between donor, procurement-related, and recipient perioperative factors, all of which contribute to the severity of the ischemia–reperfusion injury. Conversely, the ability of an allograft to recover from such injury is similarly impacted by numerous perioperative events (e.g., preoperative cardiac ischemic damage, frailty–sarcopenia, graft rejection, drug toxicity, kidney failure, or sepsis), [23–31] many of which are poorly studied. Although L-GrAFT and EASE scores accurately predict AF with excellent discrimination, these existing models have not specifically elucidated the role of perioperative events on the need for retransplantation [1, 2, 17]. Notably, this literature is exclusively based on retrospective, single-center studies which have not considered the center-volume effect, with a limited number of DCD and ECD grafts and no evaluation of the possible mitigation effect of MP technologies [1, 2]. The IMPROVEMENT project was designed to pay particular attention to these shortcomings, to capture and analyze as many of these qualitative and semi-quantitative variables as possible to build a comprehensive model that may meaningfully predict AF and guide treatment decisions regarding retransplantation. The study objectives are detailed in Table 2.

**Table 2:** Study objectives

Primary (#1)	To make a snapshot of the liver transplant activity on a global basis
Primary (#2)	To develop new algorithms for the timely prediction of Allograft Failure at 90 and 365 days using a comprehensive prospectively collected dataset based on the current clinical practice of liver transplant centers
Secondary (#3)	To validate the already existing predictive models and the newly developed algorithms on a retrospective cohort of patients from intermediate to high medium-volume transplant centers;
Secondary (#4)	To develop a novel time-based dynamic algorithm, with increasing accuracy from the 3^rd^ to 7^th^ post-operative day;
Secondary (#5)	To identify the best-time for re-transplant (after stratification according to the post-operative weeks, months, trimesters);
Secondary (#6)	To investigate differences in the incidence of allograft failure and ischemic cholangiopathy at 90 and 365 days according to standard DBD, DCD and high- risk DBD, LD donor grafts;
Secondary (#7)	To evaluate the effect of mitigation strategies on the precipitating factors of Allograft Failure at 90 and 365 days;
Secondary (#8)	To investigate the association of kinetic algorithms with development of post- LT complications (acute kidney injury, ischemic cholangiopathy, other complications);
Secondary (#9)	To identify risk factors for mortality that may contraindicate re-transplant;
Secondary (#10)	To investigate the impact of donor-to-recipient gender match on LT outcomes

## Methods

### Study design, steering committee, and participating centers

This is a multicenter, international, non-competitive, observational two-segment study.

The study segments include a first one with retrospective patient enrollment and a second one with prospective enrollment (Table 3).

**Table 3:** Study design and population

	Prospective segment (high-volume centers ≥ 65 transplants per year)	Retrospective segment (low- & intermediate- volume centers < 65 transplants per year)
Sample size	750	4200
Consecutive pts to be included	50	75
No. of Centers to be involved	15	56
Enrollment period	July ‘21–December ‘23	January ‘17–December ‘19
Data entry period	Up to January’25 (follow-up)	Up to October’24 (follow-up)
AIMs	Development/validation of NEW/previous prognostic algorithms	Validation of previous kinetic algorithms
Main parameters	AST, PLT, BIL, INR	AST, PLT, BIL, INR
Outcome data and follow-up	Incidence of AF at 90 and 365 days Comprehensive complication index Length of stay in ICU, length of stay in hospital 90d and 365d graft survival, 90d and 365d patient survival, 90d and 365d ischemic cholangiopathy-free survival Actuarial data at 12 months	Incidence of AF at 90 and 365 days Comprehensive complication index Length of stay in ICU, length of stay in hospital 90d and 365d graft survival,90d and 365d patient survival, 90d and 365d ischemic cholangiopathy-free survival Actuarial data at 48 months
Pre-operative assessment	Yes	No
Sarcopenia		Yes
Malnutrition		Yes
Cardiac risk		Yes
Renal risk		Yes
Biopsy of the graft (back table)	DBD, DCD	Only when available
Post-operative assessment of factors that favour or contraindicate re-transplant	Yes	No
Number of parameters to be recorded	297	241
Number of calculated fields	27	24

Prospective and retrospective segments

**Fig. 1:**
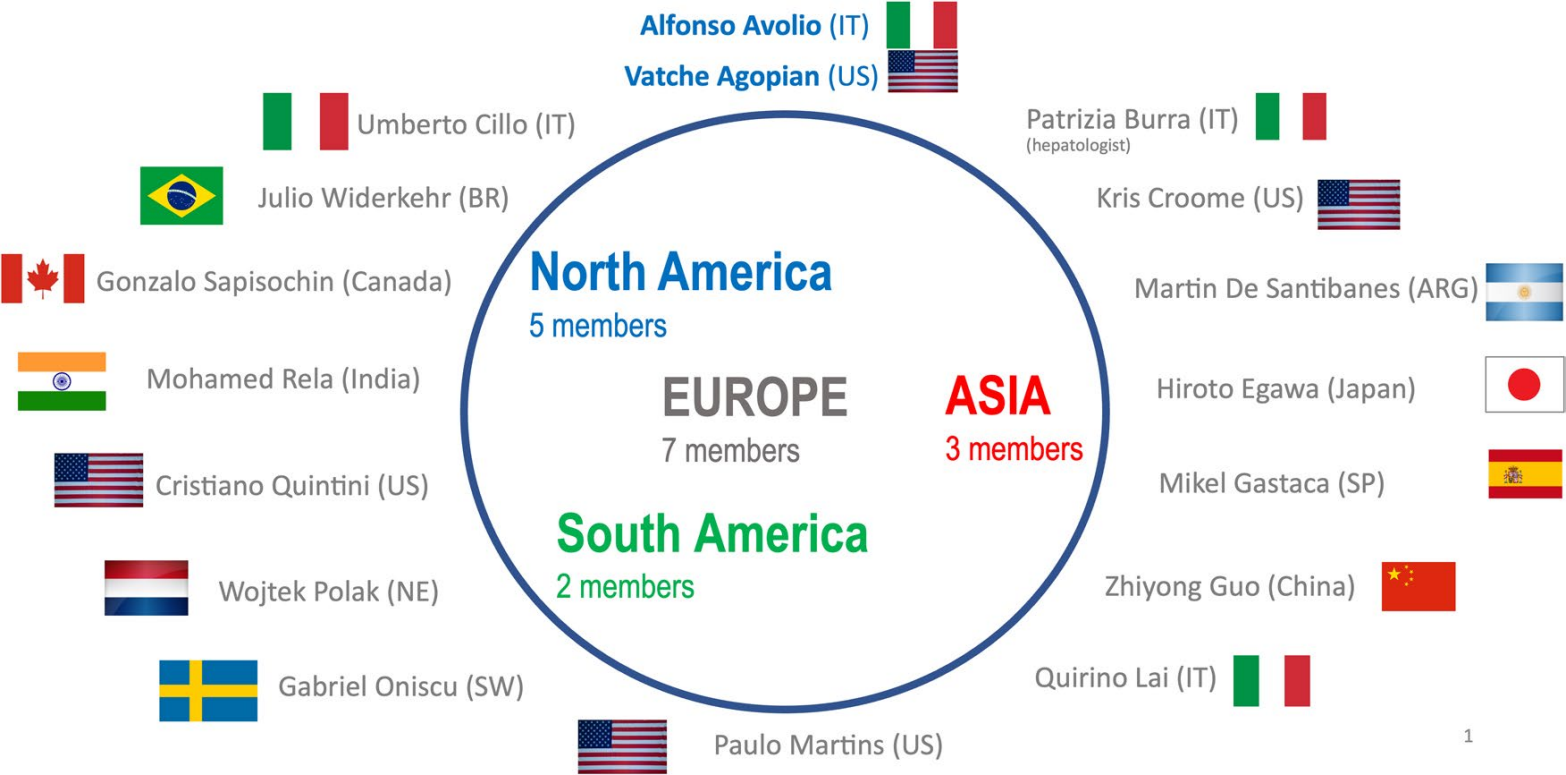
International Steering Committee

A steering committee (SC) was constituted to design the study (Fig. 1). Members were identified according to their experience on the topic and their geographic area (Europe, Americas, and Asia).

Responsibilities of the SC are:

- to select and invite other LT centers in each SC member’s geographic area;
- to supervise on the thoroughness of the data collection;
- to evaluate the robustness of the study results and make decisions on the divulgation and publication policies.

A draft of the preliminary study design and subsequent questionnaires on controversial issues were circulated among members.

Controversial issues were solved through discussion among SC members during online meetings.

**Fig. 2:**
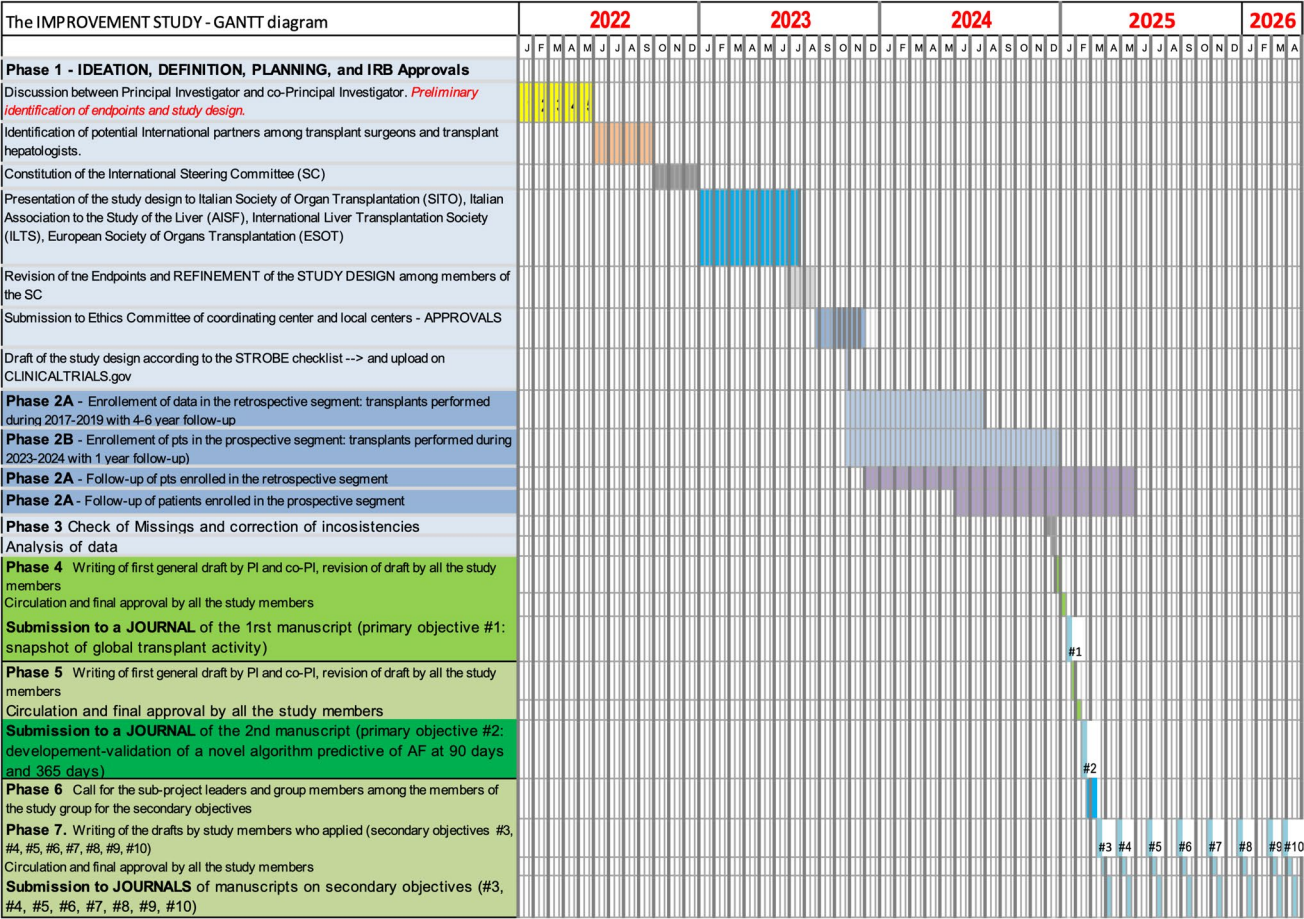
GANTT diagram

The GANTT diagram of the study is illustrated in Fig. 2.

The team leaders of participating centers amended the manuscript according to local logistical conditions, allowing a common list of parameters to be collected.

### Setting

The study will be conducted in 60 (or more) liver transplant centers representative of the global liver transplant activity (Fig. 2). The Italian liver transplant population constitutes the primary study population, while the other geographic areas (Europe without Italy, Asia, Oceania, and North and South America) will be used as a comparator. Instead, for the development of the main algorithms, the study population will be utilized as a whole.

**Fig. 3:**
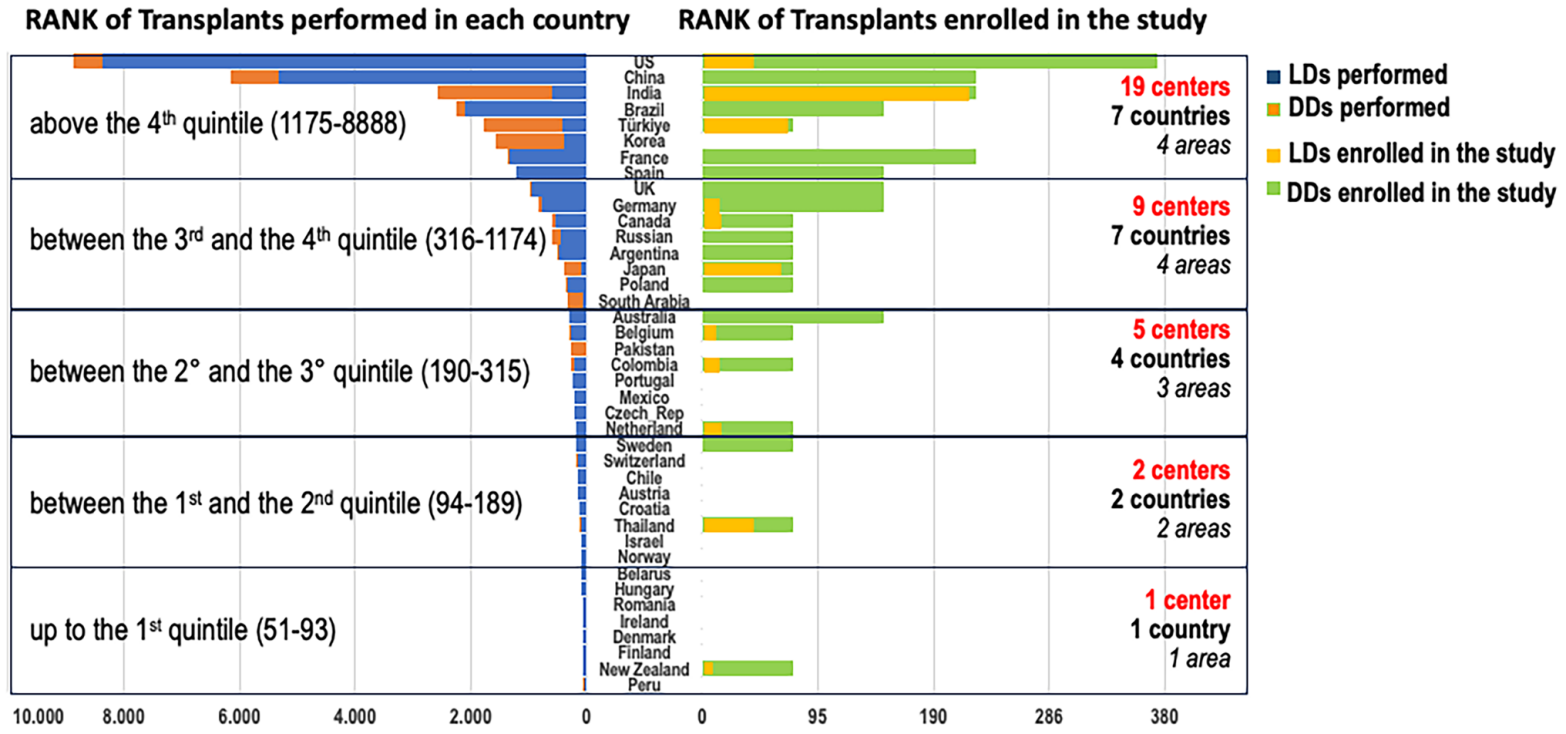
Rank of countries according to liver transplant activity and rank of enrolled transplants

Since the study aims to reflect the worldwide liver transplant activity, to balance each country contribution in the study, we used the proportions of LT activity as reported on the Global Liver Transplant Observatory [32]. This ranks the yearly number of transplants performed in various countries in decreasing order. A stratification of all cases in five classes based on quintiles was provided to parallel the number of centers to be recruited in each country. For countries in the 4^th^ and 5^th^ quintile classes, one or two centers are allowed, coherently with the 75 cases per center rule (Fig. 3), as per participation in the “Retrospective segment” of the study. Countries with < 50 cases per year are not considered.

The study will be structured in two segments, based on the enrollment modality: prospective and retrospective. Two distinct data collections will be created accordingly.

- Prospective segment. Seven hundred fifty liver transplant recipients will be enrolled in 15 high-volume centers (defined as performing ≥ 65 LT a year on average during the 2017–2019 period). Each center will enroll 50 consecutive transplants from July 2021 to December 2023 according to the inclusion criteria. For each transplant, a further 365 days will be necessary for the minimum follow-up.
- Retrospective segment. A retrospective data collection was conceived for medium–low volume centers (defined as performing < 65 LT a year), to participate in the study using the data of LTs performed between December 31, 2019, and January 1, 2017 (75 cases backward enrollment).

High-volume centers participating in the prospective segment will be allowed to enroll patients in the retrospective segment, too, voluntarily, thus enrolling 125 transplants (50 prospective and 75 retrospective transplants).

Inclusion and exclusion criteria are depicted in Table 4.

**Table 4:** Inclusion and exclusion criteria

Inclusion criteria
1. Adult cases (≥ 18 years) 2. First transplant (retransplants to be enrolled when first transplant enrolled) 3. DBD grafts 4. DCD grafts (controlled and uncontrolled) 5. DBD and DCD grafts managed by perfusion machines 6. Living donor grafts (both left lobe and right lobe grafts) transplanted into adult recipients 7. Split liver grafts (both left lobe and right lobe grafts) transplanted into adult recipients
Exclusion criteria
1. Combined grafts (e.g., liver-kidney, liver-heart, liver-pancreas, multi-visceral grafts) 2. Domino grafts 3. Heterotopic grafts 4. Double grafts 5. Cholangiocarcinoma 6. Colorectal liver metastases

Due to some concerns regarding the differences in the prevalences of AB0 incompatible grafts among different countries, these patients can be enrolled concurring to the target number of cases for each center. During the statistical analysis, we will evaluate the opportunity to include them or not in the study population, being aware of the substantial equivalence in the survival results

The software of the eCRF will check the fulfillment of the eligibility criteria and whether the target number of cases for each Center has been reached

We expect to enroll approximately 5000 patients (750 from the prospective segment and at least 4200 from the retrospective segment) (Table 5).

**Table 5:** List of participating Centers

**Italy** Fondazione Policlinico Universitario Agostino Gemelli, IRCCS; Rome, Italy Ancona hospital; Ancona, Italy AOU Policlinico consorziale di Bari; Bari, Italy ASST Papa Giovanni XXIII; Bergamo, Italy Azienda Ospedaliero-Universitaria, Policlinico di Sant'Orsola; Bologna, Italy University of Milan and National Cancer Institute, IRCCS; Milan, Italy University of Milano-Bicocca and Niguarda-Cà Granda Hospital; Milan, Italy Fondazione Cà Grande Ospedale Maggiore Policlinico, IRCCS; Milan, Italy University of Modena and Reggio Emilia; Modena, Italy Azienda Ospedaliera A. Cardarelli; Naples, Italy ISMETT IRCCS – UPMC; Palermo, Italy Azienda Ospedaliera Universitaria; Padova, Italy University of Pisa Hospital; Pisa, Italy Azienda Ospedaliera San Camillo Forlanini; Rome, Italy Sapienza University; Rome, Italy University of Rome Tor Vergata; Rome, Italy Città della Salute e della Scienza; Torino, Italy Università di Udine – ASUFC; Udine, Italy University Hospital Trust of Verona; Verona, Italy
**Europe except Italy** Cliniques Universtaires Saint-Luc; Louvein, Belgium Croix-Rousse University Hospital, Hospices Civils de Lyon; Lyon, France Beaujon Hospital, Université Paris Cité; Paris, France Regional University Hospital; Tours, France University Hospital Schleswig–Holstein; Campus Kiel, Germany Universitätsklinik Tübingen; Tubingen, Germany Erasmus University Medical Center; Rotterdam, Netherlands Surgical National Medical Research Center of Transplantation and Artificial Organs named after V.I. Shumakov, Moscow, Russia Public Central Teaching Hospital, Medical University; Warsaw, Poland University of Barcelona; Barcelona, Spain Hospital Universitario Cruces-Bilbao; Bilbao, Spain Clintec Karolinska University Hospital; Stockholm, Sweden Ankara University; Ankara, Turkey Queen Elizabeth Hospital; Birmingham, UK Newcastle Hospital NHS Foundation Trust; Newcastle, UK
**Asia** Foshan First People's Hospital; Foshan, China The First Affiliated Hospital of Sun Yat-sen University; Guangzhou, China Tongji Hospital; Wuhan, China Zhongshan People’s Hospital; Zhongshan, China Cleveland Clinic Abu Dhabi; Abu Dhabi, Emirates Dr Rela Transplant Institute and Medical center; Chennai, India Amrita Institute of Medical Sciences (Amrita Hospital); Kochi, India Max Hospital; New Delhi, India Kyoto University; Kyoto, Japan Tokyo Women's Medical University; Shizuoka, Japan Chulalongkorn University; Bangkok, Thailand Chiang Mai University; Chiang Mai, Thailand Viet Duc University Hospital; Hanoi, Vietnam
**Oceania** Austin Hospital; Melbourne, Australia University of Sydney; Sydney, Australia University of Auckland; Auckland, New Zealand
**North America** Toronto General Hospital; Toronto, Canada University of Maryland; Baltimore, USA University of Texas Southwestern Medical Center; Dallas, USA Baylor & Scott University and Medical Center; Ft. Worth & Dallas, USA Indiana University School of Medicine; Indianapolis, USA Mayo Clinic; Jacksonville, USA David Geffen School of Medicine at UCLA; Los Angeles, USA Mayo Clinic; Rochester, USA MedStar Georgetown University Hospital; Georgetown, Washington, USA University of Massachusetts; Worcester, USA
**South America** Hospital Italiano de Buenos Aires;Buenos Aires, Argentina Hospital Santa Isabel; Blumenau, Brazil University of Campinas-UNICAMP; S.Paolo, Brazil Fundation Cardio-Infantil; Bogotà, Columbia

### Study main steps

1. Creation of the study protocol and feasibility analysis (i.e., IRB approval, possibility to collect data, and resources allocation).
2. Acquisition of epidemiological data: a snapshot of the liver transplant activity in the different countries of the world.
3. Data analysis, development, and validation of prognostic algorithms.
4. Divulgation.

### Ethics and authorisations

The Ethics Committee of Fondazione Policlinico Universitario Agostino Gemelli (id 4571), Rome, and the Institutional Review Board of the University of California, Los Angeles, (respectively, the affiliations for AWA, principal investigator, and for VGA, co-principal investigator) approved the study design and the provisional study protocol. The provisional study protocol was submitted to the European Society of Organ Transplantation (ESOT), the International Liver Transplant Society (ILTS), the American Association for the study of liver disease (AASLD), the American Society of Transplantation (AST), and the Asian Society of Transplantation (AST) for suggestions and promotion among the Liver Transplant centers. The amended protocol was circulated again among the steering committee members for approval. Finally, the members submitted the study protocol to the local institutional review boards for approval.

No modification to the participating centers’ standard practice for managing LT donors and recipients is required. The study was structured according to the Guidelines for Strengthening the Reporting of Observational Studies in Epidemiology (STROBE) [33].

The study was registered on clinicaltrials.gov (NCT05289609).

### Outcome measures

Allograft failure (AF) was defined as graft failure (leading to retransplant or death) for any reason at day 90 and at day 365 after LT. This definition also captures all late-occurring AFs (also known as delayed non-function) [34, 35] We consider as AF determinants all those events potentially affecting the process of graft function recovery, independently if they were strictly associated with ischemia–reperfusion injury. Indeed, vascular (thrombosis of the hepatic artery or portal vein), biliary, toxic, and major hemodynamic events will be included, because any of them can interact with parenchymal dysfunction, affecting graft function recovery and favoring graft failure and death.

Other outcome measures are comprehensive complication index, intensive care unit length of stay, hospital length of stay (LoS), and cholangiopathy-free survival at 90 and 365 days, graft and patient survival at 90 and 365 days,

The prospective cohort dataset includes 297 data entries and 27 calculated parameters (see supplementary material 1). Variable domains are:

- identification data
- donor characteristics data
- recipient preoperative data
- graft histology data
- intraoperative data
- recipient postoperative data
- study endpoints data
- outcome and follow-up data.

The electronic Case Report Form (eCRF) was developed using the REDCap ver 10.0.21 software hosted at https://redcap-irccs.policlinicogemelli.it/. The eCRF allows easy calculation of scores including L-GrAFT and EASE scores.

The REDCAP software performs de-identification and encryption of the data.

The retrospective segment dataset includes 241 variables (supplementary material) to be collected from each center’s previously existing databases.

#### Data sources/measurement

A customized eCRF (electronic Case Report Form) was created for the study. Each local investigator will be responsible to ensure that the eCRFs is thoroughly filled in. Study data will be collected and managed using REDCap electronic data capture tools hosted at Fondazione Policlinico Universitario A. Gemelli, IRCCS (https://redcap-irccs.policlinicogemelli.it/) provided by the Research Core Facility DATA COLLECTION of the Science and Technology Park of Fondazione Policlinico Universitario A. Gemelli IRCCS (GSTeP). REDCap (Research Electronic Data Capture) is a secure, web-based application designed to support data capture for research studies, providing:

1. An intuitive interface for validated data entry;
2. Audit trails for tracking data manipulation and export procedures;
3. Automated export procedures for seamless data downloads to common statistical packages;
4. Procedures for importing data from external sources [36–39].

Only users registered as study investigators or data managers will receive a user login to access the REDCap web platform and enter/manage data. CRFs must be completed for all patients who have given informed consent. Sources of information are the physician’s patient record, hospital notes, original laboratory records, pharmacy records, and results of pathological examination. Data will be entered into the eCRF in a truthful, accurate, and timely manner.

To guarantee the highest safety and quality of data collection and management, the following were implemented:

1. RedCap was installed in GDPR compliance including database encryption and meets several security policies and user needs including compliance with 21 CFR Part 11, FISMA, HIPAA.

A two-step authentication login process has been implemented (in addition to username and password, a temporary password, sent by mail, to access the system is required);

2. The eCRF was built and will be managed according to ACCIT (Accuracy, Consistency, Completeness, Integrity and Timeliness) criteria [38, 39]. All tricks to improve data quality have been included by design such as calculated field, branching and skip logic, alert related to specific condition, and units conversion;
3. Only pseudo-anonymized data will be collected. Each center will be identified with a unique code and a progressive number will be automatically attributed by REDCap to each patient enrolled. More in depth:
  - The progressive transplant number in each center (not of the patient) and the day of the transplant will be collected.
  - The name and the surname of the patient are not recorded. However, for the convenience of the center’s operators, the first two initials of the surname and name could be optionally reported to facilitate the identification of the case during the postoperative follow-up. These initials will be visible only to the operators of the center.
4. Each center will only have access to their data. Only two operators/center can access the eCRF, one junior and one senior. Either the junior or the senior can enter and modify the records. The senior operator has two additional features:
  - Records lock after validation (it can be done in a case-by-case mode or at the end of the study according to center preference);
  - Dataset export for statistical analysis and other purposes;

NB: only de-identified data of its own center can be downloaded. De-identification allows limiting the amount of sensitive information that can be exported out of the project including one or more of the following:

1. Removing all tagged Identifier fields (tagged in Data Dictionary), unvalidated Text fields (e.g., text fields other than dates, numbers), Notes/Essay box fields.
2. Hashing the Record ID field (converts record name to an unrecognizable value).
3. Removing date and date time fields or shifting all dates by value between 0 and 364 days (shifted amount determined by algorithm for each record).

#### Potential sources of bias and pre-identified solutions

1. center participation on a voluntary basis.

Issue: some centers might withdraw their participation.

Solution: a large number of centers was planned to accommodate potential withdrawals.

2. Prospective cohort composed only by high-volume centers.

Issue: enrollment from high-volume centers might cause a loss of real-life description of the LT community situation.

Solution: only high-volume centers allow the collection of the required data to build new predictive models in a timely fashion. In addition, perfusion machines are utilized more commonly in high-volume centers. Nevertheless, data from medium-to-low volume centers will be used to validate the novel predictive model and the applicability to their clinical practice.

3. Delayed enrollment.

Issue: some high-volume centers might experience a reduction in their LT activity for various reasons.

Solution: granting extension to allow the centers to meet their enrollment expectation, up to 6 months extra.

4. Different prevalence of graft subtypes.

Issue: despite the efforts to balance the numerosity of the various LT subgroups, the number of DBD grafts remains higher than that of LD and DCD.

Solution: the preliminary sample size/power analysis calculation demonstrated that the planned enrollment allows sufficient numerosity to reveal the difference in the incidence of allograft failure at 90 and 365 days after LT.

#### Study size and power calculations

The study size was calculated to achieve adequate statistical power for the development and validation of prognostic algorithms. Calculations were independently performed by the G-Step Statistical Facility of the Fondazione Policlinico A. Gemelli IRCCS.

*1. Prior knowledge*

1.1. AF incidence

- Incidence of allograft failure (AF) at 90 days according to the L-GrAFT study (2018): 11.1% [1].
- Incidence of AF at 90 days according to the EASE score study: 6.7% [2]—Incidence of EAF according to the L-GrAFT validation study (2020) in UCLA: 7% [17].
- Incidence of AF at 90 days according to the L-GrAFT validation study (2020) in other US validation centers: 11% [17].
- Incidence of AF at 90 days according to the L-GrAFT validation study (2020) in European COPE cohort: 4% [17]. Notably, the COPE cohort consists of 222 grafts.

Research hypothesis stratified by donor category:

- 10% AF in liver transplantation with high-risk deceased donors;
- 7% AF in liver transplantation with standard deceased donors;
- 4% AF in liver transplantation with living donors.

1.2. EASE and L-GrAFT score performance as AUC (95% confidence interval)

- EASE(10) AUC = 0.87 (0.83, 0.91).
- L-GrAFT10 AUC = 0.72 (0.65, 0.78).
- L-GrAFT7 AUC = 0.78 (0.75, 0.82).

*2. L-GrAFT and EASE score algorithms validation*

Current target sample size (*n*):

- 5000 transplants overall;
- > 4125 for the retrospective study.
- 750 for the prospective study.

This estimation aims to provide the minimum sample size to achieve the baseline AUC, considering a different AF incidence for each donor category. The baseline (i.e., lowest) AUC = 0.72 of the L-GrAFT10 score is considered a minimum performance requirement to reach a conservative sample size estimation.

Assuming an overall AF rate of 6% and aiming to exclude an AUC value of 0.72, a minimum sample size of 4000 patients will result in a 95% confidence interval width of 0.072; so, if we obtain an AUC of at least 0.76, the 95% confidence interval, ranging from 0.724 and 0.796, will allow to exclude the value of 0.72.

*3. Dimension of the validation set.*

The validation set (prospective segment) will consist of 750 patients. This sample size was calculated according to Riley et al. [40] to obtain an acceptable calibration-in-the-large based on O/E, which is the ratio of the total number of observed outcome events, divided by the total number of expected (predicted) outcome events. Assuming an AF incidence rate of 0.06, 800 patients will allow to estimate an expected O/E equal to 1 with a Standard Error in log-scale of 0.14; this target will translate in a 95% confidence interval for the incidence rate ranging from 0.043 to 0.077.

### Variables and statistical analysis plan

#### Qualitative variables

All variables will be first explored by missing analysis at three levels: at center level; at country level; at geographic area level.

Histology obtained at the back-table graft preparation, or alternatively at any time before graft implantation, will constitute a key point although not a mandatory condition for the enrollment. The histology slides will be scanned at the local centers, de-identified and uploaded on the eCRF, and centrally read for research purposes.

Transplant candidates CT scan (DICOM files only) from the pre-listing work-up will be de-identified and uploaded on the eCRF and centrally read for research purposes.

The incidence and grade of ischemic cholangiopathy will be measured by means of a cholangio-MR at 10–12 months after LT (on-demand, based on clinical suspicion of ischemic cholangiopathy for all grafts, e.g., higher than twofold increase in baseline alkaline phosphatase levels). The DICOM files will be anonymised and uploaded on the eCRF and centrally read for research purposes.

#### Quantitative variables

Quantitative variables will be first explored by missing analysis at three levels: at center level, at country level, and at geographic area level.

Then, quantitative variables will be assessed by descriptive analysis in the overall population and according to three main graft types. In depth, they will be described either by mean or standard deviation (SD), whether normally distributed, or by median and interquartile range (IQR), otherwise. The Shapiro–Francia test will previously assess distribution of quantitative data.

Differences between the three main graft types ‘subgroups will be investigated using either one-way ANOVA, if normally distributed, or otherwise by the Kruskal–Wallis non-parametric test. The significance level will be set at < 0.05.

Donor characteristics, preoperative data, intraoperative data, graft histology data, and postoperative outcome-data will be also assessed as potential predictors of the main outcome, i.e., allograft survival, as well as of patients’ survival at 90 and 365 days. The characteristics mentioned above will also be implemented in algorithms to choose the best time-window within which undergoing retransplant.

The impact of donor age, graft percentage of macro-steatosis, donor warm ischemia time, donor asystolic warm ischemia time, recipient warm ischemia time, cold ischemia time, incidence of post-reperfusion syndrome, length of hospital stay, incidence of vascular thrombosis, and biliary complications (anastomotic and non-anastomotic) will be evaluated about the graft type. These characteristics will also be implemented in algorithms to evaluate the best time-window for retransplantation [14, 40–50].

The list of the definitions and abbreviations is detailed in supplementary material 2.

### Statistical analysis plan

First, a cumulative incidence of allograft failure will be calculated. To define the best time for retransplant, different strategies may be implemented, based on the collected data at study end. As first choice, we will first draw a Kaplan–Meier for each risk stratum (i.e., each graft subtype). Then, we will analyze all potential risk factors for AF in each subtype by univariate and multivariate Cox regression models. Finally, we will implement these results in a temporal algorithm based on Cox curves to identify potential time windows for retransplant. In the case of multiple drops in the initial KM curves of similar duration, we will build the algorithm so to uniform the duration of each time-window. Alternatively, we can implement a change-point analysis model derived from Kaplan–Meier estimation of the survival function followed by the least-squares estimation of the change point [51], or a wavelet analysis of change-points based on a non-parametric hazard model [52]. We could also determine the different time windows by using an extension of Glazer’s method, using a mixture of two gamma distributions, hypothesizing two or more turning points [53]. Another possibility is represented using a Bayesian approach to the problem of hazard change with unknown multiple change-points by implementing a stochastic approximation Monte Carlo algorithm for efficient calculation of the posterior distributions [54]. Particularly used in graft failure prediction. Also, a joint latent change-point class model could represent a potential way to improve the prediction of fixed time windows for retransplantation [55, 56]. All these potential applications would depend on the data distribution at the end of data collection and events observed during the follow-up period.

Notably, since the percentage of adoption of machine perfusion is expected to be one of the main differences between the two study segments, although heterogeneously worldwide, models will be adjusted according to this parameter.

i. Subgroups and interactions.

The incidence of AF at 90 and 365 as well as the incidence of death at 90 and 365 days will be calculated. Differences between the three main graft types subgroups will be investigated using either one-way ANOVA, if normally distributed, or otherwise by the Kruskal–Wallis non-parametric test. The Chi-square test will instead assess differences between qualitative variables. The significance level will be set at < 0.05.

Kaplan–Meier curves will be calculated and differences will be investigated through log-rank test.

ii. Missing data management.

A. Missing data in the calculation of the area under the curve (AUC) and the slope of the kinetic model will be addressed as follows:

1. Patients who were retransplanted or died before day 10 were excluded from the calculation of the AUC and slope because of the real impossibility to calculate the score which is by definition computed at day 10. The number of this subgroup will be reported in the flow diagram of the patients. These cases will be excluded from the patient population utilized for the development of algorithms. However, these cases will be considered for the calculation of the outcome measures (overall % of AF, % failure at 90 days, % failure at 365 days, length of stay, overall graft survival, and overall patient survival).
2. Patients who have been discharged between day 8 and 10 and do not have the day 10 determination (missing value referring to the day 10 determination of AST or Platelet count or bilirubin). In these cases, the values at day 7 will be used. The number of these cases will be reported in the flow diagram. We are aware that this approach might overestimate the value of the AUC and consequently the score’s value. However, as the number of patients without day-10 data is expected to be small, we believe that the effect will be minimal and not relevant for the purpose of the study.
3. Patients with missing data at day 2, or day 3 will be excluded, being impossible the calculation of the score. Their number will be reported in the flow diagram.
4. Patients with missing data at day 4 will be included. The AUC and the slope will be calculated using the trapezoid method not including day 4. Their number will be reported in the flow diagram.
5. Patients with missing data at day 5 will be included. The AUC and the slope will be calculated using the trapezoid method not including day 5. Their number will be reported in the flow diagram.
6. Patients with missing data at day 6 will be included. The AUC and the slope will be calculated using the trapezoid method not including day 6. Their number will be reported in the flow diagram.
7. Patients with missing data on two consecutive days (day 4 and 5, or day 5 and 6, or day 6 and 7) will be included. The AUC and the slope will be calculated using the trapezoid method not including the two consecutive missing days. Their number will be reported in the flow diagram.
8. Composite missing data (e.g., AST from one day and bilirubin from a different day) will follow the above-mentioned rule. Their number will be reported in the flow diagram.
9. Missing data in the descriptive analysis will be reported. Parameters of interest with percentage of missings higher than 8% will not be reported in tables neither will be considered for further univariate or multivariate analysis.
10. Loss to follow-up.

The count of cases lost to follow-up will be reported together with in the numbers at risk tables below the Kaplan–Meier curves.

## Discussion

This protocol refers to the first global study on liver transplantation outcomes. The study design includes several objectives based on LT practice worldwide, with centers participating in a balanced manner depending on their country’s location and activity. In addition to cross-sectional information, we expect to acquire a large volume of data to develop new models for predicting AF at different time points.

First, it will be paramount to build a new model for predicting early AF (i.e., 90-day post-LT allograft failure) based on a multicenter international comprehensive retrospective and prospective data collection. The work set by the steering committee stands on the background of the existing kinetics-based scores (L-GrAFT and EASE) constructed from a single center and a multicenter two-nation setting, respectively. Compared to these two scores, the models we aim to develop imply the advantage of a wider setting (60 centers, 21 countries, from 6 world areas) and a prospective patient enrollment. In addition to standard transplant outcome measures (graft failure and patient death at 90 and 365 days), we will pay attention to hospital and ITU length of stay, the incidence of weaning/extubation failure [57], as well as surgical complications. [58–60] Furthermore, we expect the study to allow us to develop and validate model(s) with increasing predictive accuracy from the 3rd to 7th postoperative day, making them more usable for all-day practice, bringing forward the availability of prognostic tools in the first few days after LT. We expect these tools to be able to weigh the risk of death without retransplantation and aid the decision-making processes for transplant physicians.

The presence of a retrospective study segment composed of high- and medium-volume centers guarantees a second-layer control of the applicability of previous models as well as the potential backward test of new models to real-world scenarios. The existing kinetic-based scores will be tested on the retrospective data with the aim of validating them on a large scale on a broad variety of liver transplant centers across different countries.

Second, we extended the evaluation time to 365 days, with the intent of capturing those failures occurring at a later stage. Late failure is increasingly seen nowadays due to the growing utilization of DCD and ECD grafts and the subsequent loss of grafts from ischemic cholangiopathy.

Finally, the balanced data enrollment (each center in the prospective or retrospective segment will enroll a fixed number of cases) constitutes the best solution to minimize the center-volume effect bias.

The predictive models we aim to develop would also be tailored to the changing composition of the donor pool, with an emphasis on DCD, ECD, and possible risk-mitigation strategies. Such strategies, mainly machine perfusion (MP) but also minimizing donor hepatectomy time, warm and cold ischemia time, are heterogeneously implemented in the participating centers. This will also describe the current management options and provide a snapshot of the differences in clinical practice across the centers. Since the study enrollment encompasses 7 years, including 2 years of variable LT activity due to the COVID-19 pandemic, the growing adoption of MP translates into more frequent usage in the prospective study segment. This potential bias will be taken into account in all the statistical analyses. As MP for DCD and risky DBD grafts keeps gaining momentum and more trials are coming, our study will parallel the evolving scenario of graft reconditioning and extended preservation. Differently from most studies, [61, 62], we expect to enroll more DCD and DBD cases treated with MP in both study segments. Furthermore, the global perspective of the study will allow for a deeper analysis of the mitigation effect of MP, also about center volume. Nevertheless, we are aware that since this study remains observational, novel experimental procedures such as MP-based pharmacological defatting [63] will not be incorporated.

With an all-round evaluation of the LT candidate (including frailty, nutritional status, and comorbidities), we plan to acquire data on the background condition of the recipients and stratify those risk factors that, together with intraoperative, graft histology and postoperative factors interact with each other in the generation of the transplant outcome. Gender disparity and female penalization in access to LT will also be measured and potential corrections be explored. Postoperative events and complications will be recorded, and their effect will be weighed to analyze their impact on the indication for retransplantation (e.g., when is retransplantation sustainable? How do we define the boundary of futility?) Finally, as the follow-up continues to extend, we can identify predictors of the primary disease's medium- and long-term recurrence and the development of lymphomas and epithelial neoplasms. [64].

In conclusion, the IMPROVEMENT Study will provide a large amount of data for extrapolating conclusions on various aspects in the field of liver transplant outcome prediction. It will focus on contraindications and timing of retransplantation, resource allocation, and the effect of risk-mitigation strategies, as well as the development of ischemic cholangiopathy.

## IMPROVEMENT Study-group collaborators

Salvatore Agnes —General Surgery and Transplantation Unit, Fondazione Policlinico Universitario Agostino Gemelli, IRCCS; Rome, Italy

Shekhar A. Kubal —Division of Transplant Surgery, Department of Surgery Indiana University School of Medicine; Indianapolis, USA

Roberta Angelico —HPB and Transplant Unit, Department of Surgical Sciences, University of Rome Tor Vergata; Rome, Italy

Massimo Arcerito —Department of Surgery, University of Maryland; Baltimore, USA

Arkaitz Perfecto Valero—Unidad de Cirugía Hepatobiliar y Trasplante Hepático, Hospital Universitario Cruces-Bilbao; Bilbao, Spain

Marco Biolato —Internal Medicine and Medical Transplantation Unit, Fondazione Policlinico Universitario Agostino Gemelli, IRCCS; Rome, Italy

Yang Bo —Laboratory of Organ Transplantation, Institute of Organ Transplantation, Tongji Hospital; Wuhan, China                                                                                           Mikhail Boldyrev -- National Medical Research Center of Transplantation and Artificial Organs named after V.I. Shumakov; Moscow, Russia

Eliano Bonaccorsi Riani —Service de Chirurgie et Transplantation Abdominal, Cliniques Universtaires Saint-Luc; Louvein, Belgium

Marco Bongini —General Surgery and Liver Transplantation Unit, University of Milan and National Cancer Institute, IRCCS; Milan, Italy

Jessica Bronzoni —Division of Hepatic Surgery and Liver Transplantation, University of Pisa Hospital; Pisa, Italy.

Christopher S. Chandler —Division of Liver and Pancreas Transplantation, Department of Surgery, David Geffen School of Medicine at UCLA; Los Angeles, USA

Lorenzo Cocchi —Department of HPB Surgery & Liver Transplantation, Beaujon Hospital, Université Paris Cité; Paris, France

Joris Couillerot —Department of General Surgery and Liver Transplantation, Croix-Rousse University Hospital, Hospices Civils de Lyon; Lyon, France

Cristina Elaine De Ataide —Liver Transplantation Unit, University of Campinas-UNICAMP; S.Paolo, Brazil

Riccardo De Carlis —General Surgery and Abdominal Transplantation Unit, University of Milano-Bicocca and Niguarda-CàGranda Hospital; Milan, Italy

Femke De Goeij —Department of Surgery, Erasmus University Medical Center; Rotterdam, Netherland

Andrea Della Penna —Department of General, Visceral and Transplant Surgery, Universitätsklinik Tübingen; Tubingen, Germany

Stefano Di Sandro - Hepato-pancreato-biliary Surgery and Liver Transplantation Unit, University of Modena and Reggio Emilia; Modena, Italy

Daniele Dondossola —General and Liver Transplant Surgery, Fondazione IRCCS Cà Grande Ospedale Maggiore Policlinico Milano; Milan, Italy

Emanuele Felli —Department of Digestive, Hepatobiliary and Pancreatic Surgery, Regional University Hospital; Tours, France

Daniele Ferraro —Hepato-biliary Surgery and Liver Transplant center, A.O.R.N. A. Cardarelli; Naples, Italy

Michele Finotti —Baylor Scott & White, All Saints Medical Center & Baylor University Medical Center; Ft. Worth & Dallas, USA

Francesco Frongillo General Surgery and Transplantation Unit, Fondazione Policlinico Universitario Agostino Gemelli, IRCCS; Rome, Italy

John Gundlach —Department of General, Visceral, Thoracic-, Transplant and Pediatric Surgery, University Hospital Schleswig-Holstein; Campus Kiel, Germany

Stephanie Nguyen  —MedStar Georgetown Transplant Institute, MedStar Georgetown University Hospital; Georgetown, Washington, USA

Kat Hall —Liver Transplantation unit, Austin Hospital; Melbourne, Australia

Angus Hann —Transplant Surgery, Queen Elizabeth Hospital; Birmingham, UK

Noah Kelleher —Transplant Division, Dept of Surgery, University of Massachusetts; Worcester, USA

Ninh Viet Khai —Center of Organ Transplantation, Viet Duc University Hospital; Hanoi, Vietnam

 Elvan Onur Kirimker —Liver Transplantation Unit, Department of General Surgery, Faculty of Medicine, Ankara University; Ankara, Turkey

Jagadeesh Krishnamurthy Center for Liver and Biliary Science, Max Hospital; New Delhi, India

Emilia Kruk —Transplant and Liver Surgery, Public Central Teaching Hospital, Medical University of Warsaw; Warsaw, Poland

Jacopo Lanari —General Surgey 2 Hepatobiliopancreatic Surgery and Liver Transplan Unit, Azienda Ospedaliera Universitaria; Padova, Italy

Asara Thepbunchonchai  —Division of Hepato-biliary-pancreas Surgery, Chiang Mai University; Chiang Mai, Thailand

Louise Barbier — Liver Transplant Unit, University of Auckland; Auckland, New Zealand

 Songming Li —Organ Transplant Center, The First Affiliated Hospital of Sun Yat-sen University; Guangzhou, China

Liu Ying —Department of Hepatopancreas Surgery, Foshan First People's Hospital; Foshan, China

  Laura Llado—Division of Hepatobiliary and Liver Transplantation, Department of Surgery, University of Barcelona; Barcelona, Spain

 Andrew Massutti —Liver Transplant Division, Hospital Santa IsabelM; Blumenau, Brazil

Daniela Markovic —Department of Medicine, Statistics Core, University of California, Los Angeles, USA

Fabio Melandro —Hepato-biliary-pancreatic and Liver Transplant Unit, Department of Surgery, Sapienza University; Rome, Italy

Isabel Miglior —HPB and transplant surgery, Newcastle Hospital NHS Foundation Trust; Newcastle, UK

Giovanni Moschetta —Department of General Surgery and Transplantation Unit, A.O. San Camillo Forlanini; Rome, Italy

Maria E Ramos  —Transplant Surgery, Fundation Cardioinfantil; Bogotà, Columbia

Erida Nure —General Surgery and Transplantation Unit, Fondazione Policlinico Universitario Agostino Gemelli, IRCCS; Rome, Italy

Duilio Pagano —Abdominal Transplantation, IRCCS ISMETT – UPMC; Palermo, Italy

Tommaso Partipilo —General Surgery and Transplantation Unit, Fondazione Policlinico Universitario Agostino Gemelli, IRCCS; Rome, Italy

Damiano Patrono —General Surgery 2U, Liver Transplantation Center, Azienda Ospedaliero-Universitaria Città della Salute e della Scienza di Torino; Torino, Italy

Niv Pencovich —Center for Transplantation and Clinical Regeneration, Mayo Clinic; Rochester, USA

Domenico Pinelli —Dept of Organ Failure and Transplantation, ASST Papa Giovanni XXIII; Bergamo, Italy

Jai Prasadh —Liver Transplantation Unit, University of Texas Southwestern Medical Center; Dallas, USA

Ashwin Rammohan —Institute of Liver Disease and Transplantation, Dr Rela Institute and Medical center; Chennai, India

Matteo Ravaioli —General Surgery and Transplant Unit, Azienda Ospedaliero-Universitaria di Bologna, Policlinico di Sant'Orsola; Bologna, Italy

Maria Rendina —Hepatobiliary Surgery and Liver Transplantation, AOU Policlinico consorziale di Bari; Bari, Italy

Umberto Baccarani - Liver-Kidney Transplant Unit, Università di Udine – ASUFC; Udine, Italy

Massimo Rossi —Hepato-biliary-pancreatic and Liver Transplant Unit, Department of Surgery, Sapienza University; Rome, Italy

Roberta Rossi —Hepatobiliary and Abdominal Transplantation Surgery, Ancona Hospital; Ancona, Italy

Nair Saraswathy r—Dept of GI Surgery, Amrita Institute of Medical Sciences (Amrita Hospital); Kochi, India

Patrizia Silvestri —General Surgery and Transplantation Unit, Fondazione Policlinico Universitario Agostino Gemelli, IRCCS; Rome, Italy

Qiang Sun General Surgery 1, Zhongshan People’s Hospital; Zhongshan, China

Uchida Yoichiro—Dept of Surgery, Graduate School of Medicine Kyoto University; Kyoto, Japan

Jimmy Walker Uno —Department of Hepato-Biliary-Pancreatic Surgery & Liver Transplant Unit, Hospital Italiano; Buenos Aires, Argentina

Mathias Vidgren —Division of Transplantation, Clintec Karolinska University Hospital; Stockholm, Sweden

Paola Violi —Liver Transplant Unit, University Hospital Trust of Verona; Verona, Italy

Athaya Vorasittha—Department of Surgery, Faculty of Medicine, Chulalongkorn University; Bangkok, Thailand

Claire West —Australian National Liver Transplantation Unit, Royal Prince Alfred Hospital, Faculty of Medicine and Health, University of Sydney; Sydney, Australia

## Supplementary Information

Below is the link to the electronic supplementary material.

Supplementary file1 (PDF 333 KB)

## Data Availability

Data availability statement is not appropriate here as this is a study protocol manuscript which implies that no data have been collected yet.

## Declarations

### Registration

The study was registered on clinicaltrials.gov (NCT05289609).
