# Supplementary material for: Protocol for an international multicenter, prospective, observational, non-competitive, study to validate and optimise prediction models of 90-day and 1-year allograft failure after liver transplantation: The global IMPROVEMENT Study
Source: Updates Surg. 2025 Mar 27;77(3):645–64. doi: 10.1007/s13304-025-02078-4 (PMC12226705; doi:10.1007/s13304-025-02078-4)
Supplement: Supplementary file 1 — Supplementary file1 (PDF 333 KB) [file 13304_2025_2078_MOESM1_ESM.pdf]

		PROSPECTIVE SEGMENT			RETROSPECTIVE SEGMENT	
N	SECTION	Variable Name	Variable and code's decription	Variable Type	Variable Name	N
1	ID	Progressive n° of LTX	Progressive n° of LTX according to center count (e.g. 2232)	Number	Progressive n° of LTX	1
2	ID	ID code	Hospital ID Starting 4 numbers or letters of surname - optional	NOTE	ID code	2
3	ID	TX_DATE	Date of the Transplant (or re-transplant)	DATE	TX_DATE	3
4	ID	TX_Type	1=standard: 2=Living Donor; 3=ReTransplant; 4= Heterotopic;<br>5= Combined transplant; 6=Other	Choice	TX_Type	4
5	DONOR	Age_donor	Age of the donor (years)	Number	Age_donor	5
6	DONOR	Gender_donor	Gender of the donor (phenotype)	Choice	Gender_donor	6
7	DONOR	Weight_donor	Weight of the donor (Kg or lb)	Number	Weigth_donor	7
8	DONOR	Height_donor	Height of the recipient (cm)	Number	Heigth_donor	8
9	DONOR	Graft weight	Graft weight(Back table)	Number	Graft weight	9
10	DONOR	Ethnicity_donor	1=Caucasian; 2=African-American, 3=Asian, 4=Hispanic, 5=Other	Choice	Ethnicity	10
11	DONOR	Cause_Death	Cause of Death: Trauma, Anoxia, CerebroVascular Accident, Other	Choice	Cause_Death	11
12	DONOR	Location	Local, National, Regional	Choice	Location	12
13	DONOR	SPLIT	1=no SPLIT; 2=SPLIT_right_hemiliver; 3=SPLIT_left_hemiliver	Choice	SPLIT	13
14	DONOR	AB0_incompatible	0=no AB0_incompatible; 1=AB0_incompatible	Dicho		14
15	DONOR	DCD	0=no DCD; 1=DCD	Dicho	DCD	15
16	DONOR	Controlled_DCD	0=uncontrolled_DCD; 1=controlled DCD	Dicho	Controlled_DCD	16
17	DONOR	LDTx	0=no LD; 1=LDTx (LIVING DONOR GRAFT)	Dicho	LDTx	17
18	DONOR	MP	0=no MP; 1=MP	Dicho	MP	18
19	DONOR	MP_type	1=HOPE, 2=DUAL HOPE, 3=NORMOTHERMIC; 4=IN SITU NORMOTHERMIC REGIONAL PERFUSION; 5=ISCHEMIA FREE; 6=OTHER	Choice	MP_type	19
20	DONOR	DONOR_NOTE	DONOR_NOTE	Text	DONOR_NOTE	20
21	LIVING DONOR	DONOR_TLV	Donor Total Liver Volume at CT-scan (Living donors)	Number	DONOR_TLV	21
22	LIVING DONOR	Right lobe / Left lobe	Right lobe / Left lobe	Dicho	Right lobe / Left lobe	22
23	LIVING DONOR	Anterior sector reconstruction	0=Partial or Complete MHV; 1=Segment 5 Vein; 2=Segment 8 Vein; 3=Both Segment 5 & Segment 8 vein	choice		23
24	LIVING DONOR	Recipient - Portal Modulation	0=Splenectomy; 1=Splenic Artery Ligation; 2=Collateral Ligation; 3=Hemiportocaval shunt; 4=Other	Choice	Recipient - Portal Modulation	24
25	LIVING DONOR	Portal Pressure (ppr)	Portal Pressure (post portal reperfusion) units, mmHg	Number	Portal Pressure	25
26	LIVING DONOR	Portal Pressure (ppm)	Portal Pressure (post portal modulation) units, mmHg	Number	Portal Pressure	26
27	DONOR	Donor_HT	Donor hepatectomy time<br>(minutes from start of cold flush to liver out)	Number	Donor hepatectomy time	27
~~28~~	DONOR	Donor_HT	Time from flush to liver out of the body, for the standard super-rapid retrieval technique (minutes)	Number	Donor_HT	28
29	BIOPSY	Macro Steatosis %	% of macrosteatosis	Choice	Macro Steatosis (optional)	29
30	BIOPSY	Congestion	1=None; 2=Minimal; 3=Mild; 4=Moderate; 5=Severe;	Choice	----------------------------------	
31	BIOPSY	Vacuolization	1=None; 2=Minimal; 3=Mild; 4=Moderate; 5=Severe;	Choice	----------------------------------	
32	BIOPSY	Necrosis	1=None; 2=Single Cell; 3=<30%; 4=<60%; 5=>60%;	Choice	----------------------------------	
CAL	BIOPSY	Suzuki Score	Suzuki Score	CAL	----------------------------------	
CAL	BIOPSY	Brunnel Score	1=Regular monolayer Epitelium; 2=Flattened cells; 3=Destroyed Epitelium; 4=Destroyed Epitelium + Disrupted connective tissue	Choice	----------------------------------	
33	BIOPSY	Biopsy Note	Biopsy Note	Text	Biopsy Note (optional)	30

34	Re pre-Op	LIST_DATE	DATE_OF_LIST	Date	List date	31
35	Re pre-Op	Age_recipient	Age of the recipient in years	Number	Age_recipient	32
36	Re pre-Op	Gender _recipient	Gender of the recipient (phenotype)	Choice	Gender_recipient	33
37	Re pre-Op	Weight_recipient	Weight of the recipient including ascites (Kg)	Number	Weigth_recipient	34
CAL	Re pre-Op	GRWR	Graft-to-recipient weight ratio (GRWR) CAL field	CAL	GRWR	CAL
38	Re pre-Op	Height_recipient	Height of the recipient (cm)	Number	Heigth_recipient	35
39	Re pre-Op	Ethnicity_recipient	Caucasian, African-American, Asian, Hispanic, Other	Choice	Ethnicity	36
40	Re pre-Op	Viral	1=Yes; 2=No	Dicho	Viral	37
41	Re pre-Op	Type_of_viral Indication	1=HCV; 2=HBV; 3=Other	Choice	Type_of_viral Indication	38
42	Re pre-Op	Autoimmune	1=Yes; 2=No	Dicho	Autoimmune	39
43	Re pre-Op	Cholestatic	1=Yes; 2=No	Dicho	Cholestatic	40
44	Re pre-Op	Acute Liver Failure	1=Yes; 2=No	Dicho	Acute Liver Failure	41
45	Re pre-Op	Acute on Chr liver Failure	1=Yes; 2=No	Dicho	Acute on Chronic liver Failure	42
46	Re pre-Op	Type	1=Grade 1; 2=Grade 2; 3=Grade 3;	Dicho	Type	43
47	Re pre-Op	POTUS	1=Yes; 2=No	Dicho	POTUS	44
48	Re pre-Op	NASH	1=Yes; 2=No	Dicho	NASH	45
49	Re pre-Op	NAFLD	1=Yes; 2=No	Dicho	NAFLD	46
50	Re pre-Op	MAFLD	1=Yes; 2=No	Dicho	MAFLD	47
51	Re pre-Op	Other non-neoplastic indic	1=Yes (specify) ; 2=No	Dicho	Other non-neoplastic indic	48
52	Re pre-Op	Adenoma	1=Yes; 2=No	Dicho	Adenoma	49
53	Re pre-Op	HCC	1=Yes; 2=No	Dicho	Hepatocellular carcinoma	50
54	Re pre-Op	AFT_Tx	Alpha-fetoprotein (at transplant or during previous 4 weeks)	Number	Alpha-fetoprotein	51
55	Re pre-Op	HCC_stage	0=T0; 1=T1; 2=T2; 3=T3	Choice	HCC_stage	52
56	Re pre-Op	PVT_Yerdel	0=no Trombosis; 1=Yerdel 1; 2=Yerdel 2; 3=Yerdel 3; 4=Yerdel 4	Choice	PVT_Yerdel	53
57	Re pre-Op	Bilirubin_Tx	Bilirubin at Transplant	Number	Bilirubin_Tx	54
58	Re pre-Op	Creatinine_Tx	Creatinine at Transplant	Number	Creatinine_Tx	55
59	Re pre-Op	INR_Tx	INR at Transplant	Number	INR_Tx	56
60	Re pre-Op	Na_Tx	Na at Transplant	Number	Na_Tx	57
61	Re pre-Op	Albumin_Tx	Albumin at Transplant	Number	Albumin at Transplant (optional)	58
62	Re pre-Op	Tot_Cholesterol	Total Cholesterol at Transplant (or during last 4 weeks)	Number	Tot_Cholesterol (optional)	59
63	Re pre-Op	Hb at the transplant (g/dL) units	Hb at the transplant (g/dL) units	NUMBER	Hb at the transplant (g/dL) units	60
64	Re pre-Op	Hepatic Vein Pressure Gradient (HVPG)	Hepatic Vein Pressure Gradient (HVPG) (optional) mmHg	NUMBER	Hepatic Vein Pressure Gradient (HVPG)	61
65	Re pre-Op	Tot_Lymphocyte_count	Total Lymphocyte Count at Transplant (or during last 4 weeks)	Number	Tot_Lymphocyte_count (optional)	62
CAL	Re pre-Op	CONUT score	CONUT score	CAL	CONUT score	CAL
66	Re pre-Op	Dialysis	Dialysis or CVVH at least twice in the last week 1=Yes; 2=No	Dicho	Dialysis	63
67	Re pre-Op	AFT_Tx	Alpha-fetoprotein at transplant (or previous last 4 weeks)	Number	AFT_Tx	64
CAL	Re pre-Op	MELD_Tx	MELD at Transplant (CAL field)	CAL	MELD_Tx	CAL
CAL	Re pre-Op	MELDNa_Tx	MELDNa at Transplant (CAL field)	CAL	MELD_Na	CAL
68	Re pre-Op	Dialysis	Dialysis or CVVH at least twice in the last week 1=Yes; 2=No	Dicho	Dialysis	65
69	Re pre-Op	MELD 3.0	MELD 3.0 (be sure to have inserted values for creatinine, bilirubin, INR, Na and albumin)	CAL	MELD 3.0	66
70	Re pre-Op	End stage renal disease > 3 months	End stage renal disease > 3 months. 0=Yes; 1=No;	Dicho	End stage renal disease > 3 months	67

CAL=calculated field; EI=external input field;

71	Re pre-Op	Complete loss of kidney function > 4 weeks	Complete loss of kidney function > 4 weeks. 0=Yes; 1=No;	Dicho	Complete loss of kidney function > 4 weeks	68
72	Re pre-Op	Serum creatinine	Serum creatinine. 1=up to 1.5-2x above baselin; 2= >2-3x above baseline; 3= >3x above baseline or ≥4 mg/dL	Choice	Serum creatinine	69
73	Re pre-Op	Glomerular filtration rate (GFR)	Glomerular filtration rate (GFR). 1=>25% decrease below baseline; 2=>50% decrease below baseline; 3= >75% decrease below baseline	Choice	Glomerular filtration rate (GFR)	70
74	Re pre-Op	Urine output, day 3 post-operative	Urine output, day 3 post-operative. 1=>= 0.5 mL/kg/hr x 6 hr; 2= < 0.5 mL/kg/hr x 12 hr; 3= < 0.3 mL/kg/hr x 24 hr (oliguria), or anuria x 12 hr	Choice	Urine output, day 3 post-operative	71
75	Re pre-Op	Type_of_Kidney_supp_previous_3_dd	Type of Kidney support in the previous 3 days<br>1=no; 2=CAVH (Continuous Veno-venous Hemofiltration); 3=HD (Hemodialysis)	Choice	Kidney_supp_previous_3_dd	72
CAL	Re pre-Op	RIFLE_Tx	RIFLE score at Transplant (CAL field)	CAL	RIFLE	CAL
76	Re pre-Op	PreTx-RRT	pre-transplant renal replacement therapy.<br>1=never, 2=sometimes in last month; 3=started during last 72h; 4=started during last week	Choice	PreTx-RRT (optional)	73
77	Re pre-Op	PreTx-MAS	Pre-transplant-Major Abdominal Surgery 1=Yes; 2=No	Dicho	PreTx-MAS	74
78	Re pre-Op	Ejection Fraction %	Ejection Fraction % at pre-transplant echocardiography	Number	Ejection Fraction % (optional)	75
79	Re pre-Op	Stent_Num	Coronaric Stent 0=no; 1=1 stent; 2=2 stents; 3=3 stents	Number	-----------------------------------	
80	Re pre-Op	Diabetes	Diabetes 0=Yes; 1=No;	Dicho	-----------------------------------	
81	Re pre-Op	Hypertension	Hypertension 0=Yes; 1=No;	Dicho	-----------------------------------	
82	Re pre-Op	Tobacco_years	Tobacco Pack Years (1=0-20; 2=21-40; 3= >40)	Number	-----------------------------------	
83	Re pre-Op	Family_CAD	Family History of Coronary Artery Disease<br>0=Yes; 1=No;	Dicho	-----------------------------------	
84	Re pre-Op	Personal_CAD	Personal History of Coronary Artery Disease 0=Yes; 1=No;	Dicho	-----------------------------------	
CAL	Re pre-Op	CAD-LT score	Coronary Artery Disease score	CAL		
85	Re pre-Op	Hand_strength_1	Hand grip strength (Kg) attempt 1	Number	-----------------------------------	
86	Re pre-Op	Hand_strength_2	Hand grip strength (Kg) attempt 2	Number	-----------------------------------	
87	Re pre-Op	Hand_strength_3	Hand grip strength (Kg) attempt 3	Number	-----------------------------------	
88	Re pre-Op	Sec_5_chair_sta	Time to do 5 chair stands	Number	-----------------------------------	
89	Re pre-Op	Sec_SIDE_posit	Seconds holding side position	Number	-----------------------------------	
90	Re pre-Op	Sec_SEMI-TANDEM	Seconds holding SEMI-TANDEM position	Number	-----------------------------------	
91	Re pre-Op	TANDEM_posit	Seconds holding tandem position	Number		
CAL	Re pre-Op	FRAILTY_INDEX	Frailty Index	CAL	-----------------------------------	
92	Re pre-Op	FRAILTY_assessment_date	FRAILTY_assessment_date	Date	-----------------------------------	
93	Re pre-Op	ASA_PS_class	A a patient with severe systemic disease; B patient with severe systemic disease that is a consistent threat to life; C moribund patient who is not expected to survive without the operation; D No Information (NI)	Choice	-----------------------------------	
94	Re pre-Op	Lung_support_previous_3_dd	Lung support in the previous 3 days: 1=no; 2=NIV(Non Invasive Ventilation); 3=CPAP (Continuous Positive Airway Pressure) ; 4=Mechanical Ventilation; 5=Other;	Choice	Lung_support_previous_3_dd	76
95	Re pre-Op	Type_of_Kidney_supp_previous_3_dd	Type of Kidney support in the previous 3 days<br>1=no; 2=CAVH (Continuous Veno-venous Hemofiltration); 3=HD (Hemodialysis; 4=Other	Choice	Kidney_supp_previous_3_dd	77
96	Re pre-Op	Congestive Heart Failure	Congestive Heart Failure 0=no; 1=yes	Choice	Congestive Heart Failure	78
97	Re pre-Op	Cerebral vascular accident	Cerebral vascular accident 0=no; 1=yes	Choice	Cerebral vascular accident	79
98	Re pre-Op	Peripheral vascular disease	Peripheral vascular disease 0=no; 1=yes	Choice	Peripheral vascular disease	80
99	Re pre-Op	Coronary artery disease	Coronary artery disease 0=no; 1=yes (CAL field)	Choice	Coronary artery disease	81

CAL=calculated field; EI=external input field;

100	Re pre-Op	BPCO	Chronic obstructive pulmonary dis 0=no; 1=yes	Choice	BPCO	82
101	Re pre-Op	Renal insufficiency	Renal insufficiency (continuous hemodialysis) 0=no; 1=yes	Choice	Renal insufficiency	83
102	Re pre-Op	Connective tissue disease	Connective tissue disease 0=no; 1=yes	Choice	Connective tissue disease	84
103	Re pre-Op	History of Malignancy	History of Malignancy 0=no; 1=yes	Choice	History of Malignancy	85
CAL	Re pre-Op	MCCI	Modified Charlson Comorbidity Index	CAL	MCCI	CAL
104	Re pre-Op	TIPS pre transplant	TIPS pre transplant. Transjugular intrahepatic portosystemic shunt (TIPS) is a procedure that involves inserting a stent (tube) to connect the portal veins to adjacent blood vessels that have lower pressure. This relieves the pressure of blood flowing through the diseased liver and can help stop bleeding and fluid back up. 0=no; 1=yes	Choice	TIPS pre transplant	86
105	Re pre-Op	COVID-19 status at tx	1=vaccinated pt (3 doses); 2=vaccinated pt (2 doses); 3=vaccinated pt (1 doses); 4=not vaccinated; 5=previous COVID-19 disease; 6=previous SARS-CoV-2 positivity	Choice	--------------------------------------	87
CAL	CT scan	SARCO-MODEL	Last CT scan date (upload the .zip file of the last CT-scan record; the calculation of right and left psoas muscles areas of the skeletal mass area, skeletal mass index and visceral adipose tissue will be performed by a dedicated radiologist at the coordinating center)	Date	Last CT scan	CAL
106	CT scan (Sarcopenia Evaluation on CD)	Right psoas muscle area	Right psoas muscle area (mm^2)	Number	Right psoas muscle area	88
107	CT scan (Sarcopenia Evaluation on CD)	Left psoas muscle area	Left psoas muscle area (mm^2)	Number	Left psoas muscle area	89
CAL	CT scan (Sarcopenia Evaluation on CD)	Total psoas area	Total psoas area (mm^2/m^2)	Number	Total psoas area	CAL
108	CT scan (Sarcopenia Evaluation on CD)	Skeletal muscle area	Skeletal muscle area(mm^2)	Number	Skeletal muscle area	90
CAL	CT scan (Sarcopenia Evaluation on CD)	Skeletal muscle index	Skeletal muscle index (mm^2/m^2)	Number	Skeletal muscle index	CAL
109	CT scan (Sarcopenia Evaluation on CD)	Visceral Adipose Tissue (VAT)	Visceral Adipose Tissue (VAT)	Number	Visceral Adipose Tissue (VAT)	91
110	Intra-Op	CIT	Cold Ischemia Time (hours, minutes)	Number	CIT	92
CAL	Intra-Op	DRI	Donor Risk Index	CAL		CAL
111	Intra-Op	rWIT	recipient Warm Ischemia Time (minutes)	Number	WIT	93
112	Intra-Op	Date of reperfusion	Date of venous reperfusion (for kinetic parameterers calculation)	__/__/__	Date of reperfusion (optional)	94
113	Intra-Op	Time of reperfusion	Date and Time of venous reperfusion (for kinetic parameterers calculation)	__:__	Time of reperfusion (optional)	95
114	Intra-Op	Lactete_120 minutes	Lactete 120 minutes after venous reperfusion		Lactate_120 minutes (optional)	96
115	Intra-Op	PRBC	Packed Red Blood Units transfused during surgery	Number	PRBC	97
116	Intra-Op	Cell_Saver	Cell_Saver_Use (1=yes; 2=no)	Choice	----------------------------------	
117	Intra-Op	FFP	Fresh Frozen Plasma Units transfused during surgery	Number	FFP (optional)	98
118	Intra-Op	PLT	Platelets Units transfused during surgery	Number	PLT (optional)	99
119	Intra-Op	Jump portal graft	Jump graft (Yes or not)	Choice	Jump portal graft	100
120	Intra-Op	N Arterial reconstruction	Number of arterial anastomosis (1-4)	Number	N Arterial reconstruction	101

CAL=calculated field; EI=external input field;

121	Intra-Op	Aorto-Hepatic_Conduit	Aorto-Hepatic Conduit (Yes, No)	Dicho	Aorto-Hepatic_Conduit (optional)	102
122	Intra-Op	AL	Arcuate_Legament_Management (1=preservation of gastroduodenal artery;2= release of arcuate ligament, 3=aorto-celiac anastomosis, 4=standard Center anastomosis)	Choice	----------------------------------	
123	Intra-Op	PRS	Post-Reperfusion Syndrome (none, mild, severe)	Choice	PRS (optional)	103
124	Intra-Op	Cardiac Arrest Reperfusion	CARDIAC ARREST AT REPERFUSION (1=yes; 2=no)	Choice	Cardiac Arrest Reperfusion	104
125	Intra-Op	Biliary reconstruction	Biliary anastomosis (1=duct to duct interrupted sutures; 2=duct to duct continuous sutures, 3=Roux en Y)	Choice	Biliary reconstruction	105
127	Intra-Op	Arterial lactate	Lactate (120 minutes after venous reperfusion)	Number	Lactate (optional)	
128	Intra-Op	T-Tube	T-Tube use 1=yes; 2=no	Dicho	T-Tube	106
129	Intra-Op	IntraOp_Details	Intraoperative details (VVBP, Veno-venous bypass; TPC, Temporary Porto-cava shunt, ...)	Text	IntraOp_NOTES	107
130	Post-Op	Date of 1_POD	Date of 1 POD (for kinetic parameterers calculation) usually 7.00 a.m.	__/__/__	Date of 1 POD (optional)	108
131	Post-Op	Time of reperfusion	Time of 1 POD (for kinetic parameterers calculation) usually 7.00 a.m	__:__	Time of 1 POD (optional)	109
132	Post-Op	Bilirubine_1_POD	Bilirubin at day 1 post-operative	Number	BIL_1_POD	110
133	Post-Op	Creatinine_1_POD	Creatinine at day 1 post-operative	Number	Creatinine_1_POD	111
134	Post-Op	Platelets_1_POD	Platelets at day 1 post-operative	Number	Platelets_1_POD	112
135	Post-Op	AST_1_POD	AST at day 1 post-operative	Number	AST_1_POD	113
136	Post-Op	ALT_1_POD	ALT at day 1 post-operative	Number	ALT_1_POD	114
137	Post-Op	INR_1_POD	INR at day 1 post-operative	Number	INR_1_POD	115
138	Post-Op	Lactate_1_POD	Lactate_1_POD	Number	Lactate_1_POD (optional)	116
139	Post-Op	Best EYE 1 POD	Glasgow Coma EYE 1 POD	Choice	------------	
140	Post-Op	Best VERBAL RESPONSE 1 POD	Glasgow Coma VERBAL RESPONSE 1 POD	Choice	------------	
141	Post-Op	Best MOTOR RESPON 1 POD	Glasgow Coma BEST MOTOR RESPON 1 POD	Choice	------------	
CAL	Post-Op	GLASGOW_COMA 1 POD	(CAL FIELD)	CAL	------------	
142	Post-Op	PaO2_1_POD	PaO2 day 1 post-operative	Number	------------	
143	Post-Op	FiO2_1_POD	FiO2 day 1 post-operative	Number	------------	
144	Post-Op	Mechanical Ventilation 1 POD (already recorded)	Mechanical Ventilation day 1 post-operative (already recorded) 1=yes; 2=no	Dicho	------------	
145	Post-Op	MAP70_1_POD	Mean arterial pressure or administration of vasoactive agents required at 1 POD (1=No hypotension; 2=MAP<70; 3=Dopamine<=5 or dobutamine any dose; 4=Dopamine >5, epinephrine<=0.1, norepinephrine<=0.1; 5=Dopamine>15, epinephrine>0.1 or norepinephrine>0.1	Choice	------------	
CAL	Post-Op	SOFA_1_POD	SOFA day 1 ICU – CAL field	CAL	------------	
146	Post-Op	Bilirubine_2_POD	Bilirubin at day 2 post-operative	Number	BIL_1_POD	117
147	Post-Op	Platelets_2_POD	Platelets at day 2 post-operative	Number	Platelets_2_POD	118
148	Post-Op	AST_2_POD	AST at day 2 post-operative	Number	AST_2_POD	119
149	Post-Op	ALT_2_POD	ALT at day 2 post-operative	Number	ALT_2_POD	120
150	Post-Op	INR_2_POD	INR at day 2 post-operative	Number	INR_2_POD	121
151	Post-Op	Bilirubine_3_POD	Bilirubin at day 3 post-operative	Number	Bilirubine_3_POD	122
152	Post-Op	Creatinine_3_POD	Creatinine at day 3 post-operative	Number	Creatinine_3_POD	123
153	Post-Op	Platelets_3_POD	Platelets at day 3 post-operative	Number	Platelets_3_POD	124
154	Post-Op	Blood_Albumin_3_POD	Blood Albumin at day 3 post-operative	Number	------------	
155	Post-Op	Urine_Albumin_3_POD	Urine albumin at day 3 post-operative	Number	------------	
CAL	Post-Op	Albumin/creatin ratio 3 POD	Albumin to creatinine ratio at 3 post-operative	CAL	------------	
156	Post-Op	Noradrenalin dose	Noradrenalin dose at day 3 post-operative	Number	--------------------------------	

CAL=calculated field; EI=external input field;

			(if patient does not require noradrenalin please write 0)			
157	Post-Op	AST_3_POD	AST at day 3 post-operative	Number	AST_3_POD	125
158	Post-Op	ALT_3_POD	ALT at day 3 post-operative	Number	AST_3_POD	126
159	Post-Op	INR_3_POD	INR at day 3 post-operative	Number	INR_3_POD	127
160	Post-Op	Lactate_3_POD	Lactate at day 3 post-operative	Number	Lactate_3_POD (optional)	128
161	Post-Op	Ejection Fraction	Ejection fraction at day 3 post-operative	Number	------------	
162	Post-Op	Noradrenaline dose	Noradrenaline dose at day 3 post-operative	Number	------------	
163	Post-Op	Best EYE 3 POD	Glasgow Coma EYE 3 POD	Choice	------------	
164	Post-Op	Best VERB RESP 3 POD	Glasgow Coma VERBAL RESPONSE 3 POD	Choice	------------	
165	Post-Op	Best MOTOR RESP 3 POD	Glasgow Coma BEST MOTOR RESPONSE 3 POD	Choice	------------	
CAL	Post-Op	GLASGOW_SC 3 POD	(CAL FIELD)	CAL	------------	
166	Post-Op	PaO2_3_POD	PaO2 day 3 post-operative	Number	------------	
167	Post-Op	FiO2_3_POD	FiO2 day 3 post-operative	Number	------------	
168	Post-Op	Mechanical Ventilation 3 POD (already recorded)	Mechanical Ventilation day 3 post-operative (already recorded) 0=yes; 1=no	Dicho	------------	
169	Post-Op	MAP70_3_POD	Mean arterial pressure or administration of vasoactive agents required at 3 POD (1=No hypotension; 2=MAP<70; 3=Dopamine<=5 or dobutamine any dose; 4=Dopamine >5, epinephrine<=0.1, norepinephrine<=0.1; 5=Dopamine>15, epinephrine>0.1 or norepinephrine>0.1	Choice	------------	
CAL	Post-Op	SOFA_3_POD	SOFA day 3 ICU – CAL field	CAL	------------	
170	Post-Op	Serum creatinine	Serum creatinine, day 3 post-operative. 1=up to1.5-2x above baseline; 2=>2-3x above baseline; 3= >3x above baseline or ≥4 mg/dL	Choice	Serum creatinine	129
171	Post-Op	Glomerular filtration rate (GFR)	Glomerular filtration rate (GFR), day 3 post-operative. 1= >25% decrease below baseline; 2= >50% decrease below baseline;3= >75% decrease below baseline	Choice	Glomerular filtration rate (GFR)	130
172	Post-Op	Urine output	Urine output, day 3 post-operative. 1=>= 0.5 mL/kg/hr x 6 hr; 2= < 0.5 mL/kg/hr x 12 hr; 3= < 0.3 mL/kg/hr x 24 hr (oliguria), or anuria x 12 hr	Choice	Urine output	131
CAL	Post-Op	RIFLE score_3_POD	RIFLE score at day 3 post-operative	CAL	------------	
173	Post-Op	RIFLE criteria_3_POD	RIFLE criteria at day 3 post-operative	CAL	------------	
174	Post-Op	Bilirubin_4_POD	Bilirubin at day 4 post-operative	Number	Bilirubine_4_POD	132
175	Post-Op	Platelets_4_POD	Platelets at day 4 post-operative	Number	Platelets_4_POD	133
CAL	Post-Op	AST_4_POD	AST at day 4 post-operative	Number	AST_4_POD	CAL
176	Post-Op	ALT_4_POD	ALT at day 4 post-operative	Number	ALT_4_POD	134
177	Post-Op	INR_4_POD	INR at day 4 post-operative	Number	INR_4_POD	135
178	Post-Op	Bilirubine_5_POD	Bilirubin at day 5 post-operative	Number	Bilirubine_5_POD	136
179	Post-Op	Creatinine_5_POD	Creatinine at day 5 post-operative	Number	Creatinine_5_POD	137
180	Post-Op	Platelets_5_POD	Platelets at day 5 post-operative	Number	Platelets_5_POD	138
181	Post-Op	Serum creatinine	Serum creatinine, day 3 post-operative. 1=up to1.5-2x above baseline; 2=>2-3x above baseline; 3= >3x above baseline or ≥4 mg/dL	Choice	Serum creatinine	139
182	Post-Op	Glomerular filtration rate (GFR)	Glomerular filtration rate (GFR), day 3 post-operative. 1= >25% decrease below baseline; 2= >50% decrease below baseline;3= >75% decrease below baseline	Choice	Glomerular filtration rate (GFR)	140
183	Post-Op	Urine output	Urine output, day 3 post-operative. 1=>= 0.5 mL/kg/hr x 6 hr; 2= < 0.5 mL/kg/hr x 12 hr; 3= < 0.3 mL/kg/hr x 24 hr (oliguria), or anuria x 12 hr	Choice	Urine output	141
184	Post-Op	AST_5_POD	AST at day 5 post-operative	Number	AST_5_POD	142
185	Post-Op	ALT_5_POD	ALT at day 5 post-operative	Number	ALT_5_POD	143
186	Post-Op	INR_5_POD	INR at day 5 post-operative	Number	INR_5_POD	144

CAL=calculated field; EI=external input field;

187	Post-Op	Lactate_5_POD	Lactate at day 5 post-operative	Number	Lactate_5_POD (optional)	145
188	Post-Op	Serum creatinine	Serum creatinine, day 3 post-operative. 1=up to1.5-2x above baseline; 2=>2-3x above baseline; 3= >3x above baseline or ≥4 mg/dL	Choice	Serum creatinine	146
189	Post-Op	Glomerular filtration rate (GFR)	Glomerular filtration rate (GFR), day 3 post-operative. 1= >25% decrease below baseline; 2= >50% decrease below baseline;3= >75% decrease below baseline	Choice	Glomerular filtration rate (GFR)	147
190	Post-Op	Urine output	Urine output, day 3 post-operative. 1=>= 0.5 mL/kg/hr x 6 hr; 2= < 0.5 mL/kg/hr x 12 hr; 3= < 0.3 mL/kg/hr x 24 hr (oliguria), or anuria x 12 hr	Choice	Urine output	148
CAL	Post-Op	RIFLE score_5_POD	RIFLE score at day 5 post-operative	CAL	------------	
191	Post-Op	RIFLE criteria_5_POD	RIFLE criteria at day 5 post-operative	CAL	------------	
192	Post-Op	Bilirubine_6_POD	Bilirubin at day 6 post-operative	Number	Bilirubine_6_POD	149
193	Post-Op	Platelets_6_POD	Platelets at day 6 post-operative	Number	Platelets_6_POD	150
194	Post-Op	AST_6_POD	AST at day 6 post-operative	Number	AST_6_POD	151
195	Post-Op	ALT_6_POD	ALT at day 6 post-operative	Number	ALT_6_POD	152
196	Post-Op	INR_6_POD	INR at day 6 post-operative	Number	INR_6_POD	153
197	Post-Op	Bilirubine_7_POD	Bilirubin at day 7 post-operative	Number	Bilirubine_7_POD	154
198	Post-Op	Creatinine_7_POD	Creatinine at day 7 post-operative	Number	Creatinine_7_POD	155
199	Post-Op	Platelets_7_POD	Platelets at day 5 post-operative	Number	Platelets_7_POD	156
200	Post-Op	AST_7_POD	AST at day 7 post-operative	Number	AST_7_POD	157
201	Post-Op	ALT_7_POD	ALT at day 7 post-operative	Number	ALT_7_POD	158
202	Post-Op	INR_7_POD	INR at day 7 post-operative	Number	INR_7_POD	159
203	Post-Op	Serum creatinine	Serum creatinine, day 3 post-operative. 1=up to1.5-2x above baseline; 2=>2-3x above baseline; 3= >3x above baseline or ≥4 mg/dL	Choice	Serum creatinine	160
204	Post-Op	Glomerular filtration rate (GFR)	Glomerular filtration rate (GFR), day 3 post-operative. 1= >25% decrease below baseline; 2= >50% decrease below baseline;3= >75% decrease below baseline	Choice	Glomerular filtration rate (GFR)	161
205	Post-Op	Urine output	Urine output, day 3 post-operative. 1=>= 0.5 mL/kg/hr x 6 hr; 2= < 0.5 mL/kg/hr x 12 hr; 3= < 0.3 mL/kg/hr x 24 hr (oliguria), or anuria x 12 hr	Choice	Urine output	162
CAL	Post-Op	RIFLE score_7_POD	RIFLE score at day 7 post-operative	CAL	------------	
206	Post-Op	RIFLE criteria_7_POD	RIFLE criteria at day 7 post-operative	CAL	------------	
207	Post-Op	Bilirubine_8_POD	Bilirubin at day 8 post-operative	Number	Bilirubine_8_POD	163
208	Post-Op	Platelets_8_POD	Platelets at day 8 post-operative	Number	Platelets_8_POD	164
209	Post-Op	AST_8_POD	AST at day 8 post-operative	Number	AST_8_POD	165
210	Post-Op	ALT_8_POD	ALT at day 8 post-operative	Number	ALT_8_POD	166
211	Post-Op	INR_8_POD	INR at day 8 post-operative	Number	INR_8_POD	167
212	Post-Op	Bilirubine_9_POD	Bilirubin at day 9 post-operative	Number	Bilirubine_9_POD	168
213	Post-Op	Platelets_9_POD	Platelets at day 9 post-operative	Number	Platelets_9_POD	169
214	Post-Op	AST_9_POD	AST at day 9 post-operative	Number	AST_9_POD	170
215	Post-Op	ALT_9_POD	ALT at day 9 post-operative	Number	ALT_9_POD	171
216	Post-Op	INR_9_POD	INR at day 9 post-operative	Number	INR_9_POD	172
217	Post-Op	Bilirubine_10_POD	Bilirubin at day 10 post-operative	Number	Bilirubine_10_POD	173
218	Post-Op	Platelets_10_POD	Platelets at day 10 post-operative	Number	Platelets_10_POD	174
219	Post-Op	AST_10_POD	AST at day 10 post-operative	Number	AST_10_POD	175
220	Post-Op	ALT_10_POD	ALT at day 10 post-operative	Number	ALT_10_POD	176
221	Post-Op	INR_10_POD	INR at day 10 post-operative	Number	INR_10_POD	177
CAL	Post-Op	L-GrAFT-7_score	LGraft-7 score	CAL	L-GrAFT-7_score	CAL
CAL	Post-Op	L-GrAFT-10_score	LGraft-10 score	CAL	L-GrAFT-10_score	CAL

CAL=calculated field; EI=external input field;

222	Post-Op	Thrombosis_type	Thrombosis type; 1=no thrombosis; 2=artery; 3=portal vein; 4=cava; 5=artery+portal vein; 6=artery+portal vein+cava	Choice	Thrombosis_type	178
CAL	Post-Op	EASE_score	EASE score	CAL	EASE_score	CAL
223	Post-Op	Respiratory complication	1=Extubation failure; 2=Respiratory failure	Choice	----------------	
224	Post-Op	Days of invasive ventilation	Days of invasive ventilation (sum of different periods in case of one or more days without invasive ventilation)	Number	Days of invasive ventilation (optional)	179
225	Complication	Did the patient have any complication	Did the patient have any major/minor post-operative complication?<br>1=yes; 2=no	Dicho	Did the patient have any complication	180
226	Complication	Bleeding complication	Bleeding complication required surgical intervention 1=yes; 2=no	Dicho	Bleeding complication	181
227	Complication	Endovascular treatment	0=no; 1=yes	Choice	Endovascular treatment	182
228	Complication	Endovascular treat details	notes	Note	Endovascular treat details	183
229	Complication	Ascites requiring TIPS	Transjugular intrahepatic portosystemic shunt (TIPS) is a procedure that involves inserting a stent (tube) to connect the portal veins to adjacent blood vessels that have lower pressure. This relieves the pressure of blood flowing through the diseased liver and can help stop bleeding and fluid back up. 0=no; 1=yes	Dicho	Ascites requiring TIPS	184
230	Complication	Antithrombotic prophylaxis	0=no prophylaxis; 1=.i.v. heparin; 2=low weight subcutaneous heparin; 3=unfractionated subcutaneous heparin 3=other	Choice	Anti-thrombotic prophylaxis	185
231	Complication	Pre-operative_INDUCTION	0=no induction; 1=Simulect; 2=other	Choice	Pre-operative_INDUCTION	186
232	Complication	Immunosuppression 1 month	1.Tacrolimus; 2.Tacrolimus-->Everolimus; 3.Tacrolimus+MMF; 4=other	Choice	Immunosuppression 1 month	187
233	Complication	Planned Use of steroids DURING POST-OPERATIVE period	Planned Use of steroids DURING POST-OPERATIVE period<br>(treatment of rejection is reported in the following field)	Choice	Planned Use of steroids DURING POST-OPERATIVE period	188
234	Complication	Rejection_treatment 1 month	1.Steroids boluses; 2. Steroids recycle; 3. Anti-tymocyte globulin; 4. Simulect; 5=other	Choice	Rejection treatment 1 month	189
235	Complication	Infection_sites	1=liver; 2=abdominal extra-liver; 3=lung; 4=heart; 5=other	Choice	Infection_sites (optional)	190
236	Complication	Bacteremia_episodes	Number_of_bacteremia_episodes (0 to N)	Number	Bacteremia_episodes (optional)	191
237	Complication	Sepsis_episodes	0=no; 1=yes	Dicho	Sepsis_episodes (optional)	192
238	Complication	Septic_shock	0=no; 1=yes	Dicho	Septic_shock (optional)	193
239	Complication	Viral_infection	0=no; 1=yes	Dicho	Viral_infection (optional)	194
240	Complication	Viral_infection_note	Viral_infection_note	Note	Viral_infection_note (optional)	195
241	Complication	Brief descript of PO course	Brief description of complicated postoperative course (note)	Text	Brief descript of PO course	196
242	Complication	Type of complication		Choice	Type of complication	197
CAL	Complication	Clavien-Dindo	1=1; 2=2; 3=3A; 4=3B; 5=4A; 6=4B	Choice	Clavien-Dindo	CAL
243	Complication	Type of complication		Choice	Type of complication	198
244	Complication	Clavien-Dindo	1=1; 2=2; 3=3A; 4=3B; 5=4A; 6=4B	Choice	Clavien-Dindo	199
CAL	Complication	CCI	Comprehensive Complication Index	Choice	CCI	CAL
245	Endpoint Evaluation at 90 and 365 days	Chol_90_DAYS_RESULT	CT, MRI, ERCP or PC cholangiography, up to 90 days<br>1=normal; 2=anastomotic stenosis; 3=non anastomotic stenosis; 4=multiple non anastomotic stenosis; 5=Normal hepatic artery; 6=hepatic artery stenosis; 7=hepatic artery thrombosis; 8=hepatic abscess (one); 9=hepatic abscess (several)	Choice	Chol_90_DAYS_RESULT	200
246	EE at 90 & 365	Diagnosis obtained by	1=TC; 2=MRI; 3=cholangiogram trans-T tube 4=ERCP; 5=PC	Choice	Diagnosis obtained by (optional)	201
247	EE at 90 & 365	MRI Chol_90_DAYS	1=NORMAL, 2=DIFFUSE NECROSIS; 3=MULTI-FOCAL PROGRESSIVE, 4=CONFLUENCE DOMINANT; 5=MINOR FORM	Choice	MRChol_90_DAYS (optional)	202
248		Please upload significant images (CT, MRI, ERCP, PC cholangiogram) OPTIONAL	Please upload significant images (CT, MRI, ERCP, PC cholangiogram) OPTIONAL		Please upload significant images (CT, MRI, ERCP, PC cholangiogram)	203

CAL=calculated field; EI=external input field;

					OPTIONAL	
249	EE at 90 & 365	MRI_chol_90_DAYS_TEXT	Brief description of MRI_cholangiogram	Note	MRI_chol_90_DAYS_TEXT (optional)	204
250	EE at 90 & 365	STENT_90_DAYS	1=yes; 2=no	Dicho	STENT_90_DAYS (optional)	205
251	EE at 90 & 365	STENT_#_90_DAYS_	NUMBER OF STENTS	Number	STENT_#_90_DAYS_ (optional)	206
252	EE at 90 & 365	AP_90_DAYS	ALKALINE_PHOSFATASIS_AT_90_DAYS	Number	AP_90_DAYS	207
253	EE at 90 & 365	gammaGT_90_MO	gammaGT at 90 days	Number	gammaGT_90_MO	208
254	EE at 90 & 365	MRI Chol_12_MO_RESULT	CT, MRI, ERCP or PC cholangiography, up to 12 months<br>1=normal; 2=anastomotic stenosis; 3=non anastomotic stenosis; 4=multiple non anastomotic stenosis; 5=Normal hepatic artery; 6=hepatic artery stenosis; 7=hepatic artery thrombosis; 8=hepatic abscess (one); 9=hepatic abscess (several)	Text	Chol_12_MO_RESULT (optional)	209
255	EE at 90 & 365	Diagnosis obtained by	1=TC; 2=MRI; 3=cholangiogram trans-T tube 4=ERCP; 5=PC	Choice	Diagnosis obtained by	210
256	EE at 90 & 365	MRI Chol_12_MO_DAYS	1=NORMAL, 2=DIFFUSE NECROSIS; 3=MULTI-FOCAL PROGRESSIVE, 4=CONFLUENCE DOMINANT; 5=MINOR FORM	Choice	MRI Chol_12_MO_DAYS (optional)	211
		Please upload significant images (CT, MRI, ERCP, PC cholangiogram) OPTIONAL	Please upload significant images (CT, MRI, ERCP, PC cholangiogram) OPTIONAL		Please upload significant images (CT, MRI, ERCP, PC cholangiogram) OPTIONAL	212
257	EE at 90 & 365	MRI_chol_12_MO_TEXT	Brief description of MRI_cholangiogram	Text	MRI_chol_12_MO_TEXT	213
258	EE at 90 & 365	STENT_12_MO	1=yes; 2=no	Dicho	STENT_12_MO	214
259	EE at 90 & 365	STENT_#_12_MO	NUMBER OF STENTS at 12 months	Number	STENT_#_12_MO	215
260	EE at 90 & 365	AP_12_MO	ALKALINE_PHOSFATASIS_AT_12 months	Number	AP_12_MO	216
261	EE at 90 & 365	gammaGT_12_MO	gammaGT at 12 months	Number	gammaGT_12_MO	217
262	OUTCOME	ICU_stay	days	Number	ICU_stay	218
263	OUTCOME	Hospital_stay	days	Number	Hospital_stay	219
264	OUTCOME	Last FU day	Last follow-up day (date)	Date	Last FU day	220
265	OUTCOME	Graft Failure FU	Graft Failure at last follow-up day available 0=no failure; 1=failure	Dicho	Graft Failure FU	221
266	OUTCOME	Intraoperative	1=bleeding; 2=post reperfusion syndrome; 3=cardiac arrest shock	Choice	Intraoperative	222
267	OUTCOME	PO CV event	Postoperative Cardiovascular events 1=yes; 2=no	Dicho	PO CV event	223
268	OUTCOME	PNF	1=yes; 2=no	Dicho	PNF	224
269	OUTCOME	Post operative bleeding	1=yes; 2=no	Dicho	Post operative bleeding	225
270	OUTCOME	Thrombosis	1=artery (main branch); 2=portal vein; 3=cava	Choice	Thrombosis	226
271	OUTCOME	Infection	(including septic shock) 1=Intra-hepatic; 2=Extra-hepatic	Dicho	Infection	227
272	OUTCOME	Cerebral complication	1=yes; 2=no	Dicho	Cerebral complication	228
273	OUTCOME	Rejection unresponsive	Rejection unresponsive to repeat treatment 1=yes; 2=no	Dicho	Rejection unresponsive	229
274	OUTCOME	Ischemic Cholangiopathy	Ischemic Cholangiopathy (multiple bil structures – no thrombosis) 0=yes; 1=no	Dicho	Ischemic Cholangiopathy	230
275	OUTCOME	Recurrence of prim dis	1=yes; 2=no	Dicho	Recurrence of prim disease	231
276	OUTCOME	De novo neoplasm	1=solid neoplasm; 2=hematological	Dicho	De novo neoplasm	232
277	OUTCOME	Other	Note	Text	Other	233
278	OUTCOME	Main cause of graft failure		Choice	Main cause of graft failure	234
279	OUTCOME	Patient death	1=Death; 2=Alive	Dicho	Patient death	235
280	OUTCOME	Intraoperative	1=Bleeding; 2= Post-reperfusion syndrome<br>3=Cardiac arrest/shock	Choice		

CAL=calculated field; EI=external input field;

281	OUTCOME	Postoperative cardiovascular event (excluding septic shock)	1=yes; 2=no	Dicho		
282	OUTCOME	PNF	1=yes; 2=no	Dicho		
283	OUTCOME	Postoperative bleeeding	1=yes; 2=no	Dicho		
284	OUTCOME	Thrombosis	1=Artery (main branch); 2= Portal vein 3=Cava	Choice		
285	OUTCOME	Infection (including septic shock)	1= Intra-hepatic; 2=Extra-hepatic	Choice		
286	OUTCOME	Cerebral complications	1=yes; 2=no	Dicho		
287	OUTCOME	Rejection	1=yes; 2=no	Dicho		
288	OUTCOME	Ischemic Cholangiopathy (multiple biliary structures in absence of arterial thrombosis)	1=yes; 2=no	Dicho		
289	OUTCOME	Recurrence of primary disease	1=yes; 2=no	Dicho		
290	OUTCOME	De novo neoplasm	1=Solid neoplasm; 2= Hematological	Choice		
291	OUTCOME	Other	Brief description			
292	OUTCOME	Main cause of death	1=Intraoperative Bleeding; 2= Intraoperative Post-reperfusion syndrome; 3= Intraoperative Cardiac arrest/shock, 4= Postoperative cardiovascular event (excluding septic shock); 5= PNF; 6= Postoperative bleeding; 7= Thrombosis: Artery (main branch); 8= Thrombosis :Portal vein; 9= Thrombosis :Cava; 10= Intra-hepatic Infection; 11=Extra-hepatic Infection; 12= Cerebral complications; 13= Rejection ; 14= schemic Cholangiopathy (multiple biliary structures in absence of arterial thrombosis) ; 15= Recurrence of primary disease; 16= De novo neoplasm:Solid neoplasm ; 17= De novo neoplasm:Hematological.	Choice	Main cause of death	236
293	OUTCOME	Last follow-up day (day of death)	Last follow-up day (day of death)Date	Date	Last follow-up day (day of death)	237
294	OUTCOME	Retransplanted	1=yes; 2=no	Dicho	Retransplanted	238
295	OUTCOME	Retransplanted date	Retransplanted data (date)	Date	Retransplanted date	239
296	OUTCOME	Contraindication to ReTx	1= Not applicable (favorable outcome) 2=Hemodynamic instability; 3=sepsis; 4=septic shock; 5=malnutrition; 6=frailty; 7=sarcopenia; 8=cardiac insufficiency; 9=renal insufficiency; 10=technical reason; 11=other (please specify)	Choice	Contraindication to ReTx (optional)	240
297	OUTCOME	Registration problems	Registration problems (note)	Text	Registration problems	241

CAL=calculated field; EI=external input field;
